# Proteomic quantification of receptor tyrosine kinases involved in the development and progression of colorectal cancer liver metastasis

**DOI:** 10.3389/fonc.2023.1010563

**Published:** 2023-02-20

**Authors:** Areti-Maria Vasilogianni, Zubida M. Al-Majdoub, Brahim Achour, Sheila Annie Peters, Amin Rostami-Hodjegan, Jill Barber

**Affiliations:** ^1^ Centre for Applied Pharmacokinetic Research, Division of Pharmacy and Optometry, School of Health Sciences, University of Manchester, Manchester, United Kingdom; ^2^ Department of Biomedical and Pharmaceutical Sciences, College of Pharmacy, University of Rhode Island, Kingston, RI, United States; ^3^ Translational Quantitative Pharmacology, BioPharma, R&D Global Early Development, Merck KGaA, Darmstadt, Germany; ^4^ Translational Medicine and Clinical Pharmacology, Boehringer Ingelheim Pharma GmbH & Co., KG, Ingelheim am Rhein, Germany; ^5^ Simcyp Division, Certara Inc., Sheffield, United Kingdom

**Keywords:** liver cancer, colorectal cancer, metastasis, receptor tyrosine kinases, QconCAT, systems biology of cancer

## Abstract

**Introduction:**

Alterations in expression and activity of human receptor tyrosine kinases (RTKs) are associated with cancer progression and in response to therapeutic intervention.

**Methods:**

Thus, protein abundance of 21 RTKs was assessed in 15 healthy and 18 cancerous liver samples [2 primary and 16 colorectal cancer liver metastasis (CRLM)] matched with non-tumorous (histologically normal) tissue, by a validated QconCAT-based targeted proteomic approach.

**Results:**

It was demonstrated, for the first time, that the abundance of EGFR, INSR, VGFR3 and AXL, is lower in tumours relative to livers from healthy individuals whilst the opposite is true for IGF1R. EPHA2 was upregulated in tumour compared with histologically normal tissue surrounding it. PGFRB levels were higher in tumours relative to both histologically normal tissue surrounding tumour and tissues taken from healthy individuals. The abundances of VGFR1/2, PGFRA, KIT, CSF1R, FLT3, FGFR1/3, ERBB2, NTRK2, TIE2, RET, and MET were, however, comparable in all samples. Statistically significant, but moderate correlations were observed (Rs > 0.50, p < 0.05) for EGFR with INSR and KIT. FGFR2 correlated with PGFRA and VGFR1 with NTRK2 in healthy livers. In non-tumorous (histologically normal) tissues from cancer patients, there were correlations between TIE2 and FGFR1, EPHA2 and VGFR3, FGFR3 and PGFRA (p < 0.05). EGFR correlated with INSR, ERBB2, KIT and EGFR, and KIT with AXL and FGFR2. In tumours, CSF1R correlated with AXL, EPHA2 with PGFRA, and NTRK2 with PGFRB and AXL. Sex, liver lobe and body mass index of donors had no impact on the abundance of RTKs, although donor age showed some correlations. RET was the most abundant of these kinases in non-tumorous tissues (~35%), while PGFRB was the most abundant RTK in tumours (~47%). Several correlations were also observed between the abundance of RTKs and proteins relevant to drug pharmacokinetics (enzymes and transporters).

**Discussion:**

DiscussionThis study quantified perturbation to the abundance of several RTKs in cancer and the value generated in this study can be used as input to systems biology models defining liver cancer metastases and biomarkers of its progression.

## Introduction

Cancer is a leading cause of death globally with increasing incidence (1). Colorectal cancer (CRC) is the third most common and the second most lethal type of cancer (2). It mainly metastasizes to the liver, followed by the lungs, distant lymph nodes, and peritoneum (3), with approximately one fourth of the patients having liver metastasis at the initial diagnosis of primary cancer, and half of the patients having liver metastasis during the course of the disease (4). Primary liver cancer has also high rates of mortality, and its main types are hepatocellular carcinoma (HCC) and intrahepatic cholangiocarcinoma (ICC) (2). Although surgical resection of liver cancer (primary or secondary) is the ideal intervention for treatment and long-term survival, this is not always possible and other methods are used, including chemotherapy, biologic therapy, radio-embolization, and radiofrequency ablation with the aim of suppressing the tumour (5, 6).

Protein kinases are important regulators of cell signalling and almost half of the human kinases can be mapped to known diseases, including cancer. Kinases may be mutated or dysregulated leading to perturbed signalling pathways, which makes kinases important disease biomarkers and pharmacological targets for cancer treatment (7, 8). Kinase inhibitors are widely used in oncology, with most new FDA approved anti-cancer molecular entities in the period 2011-2017 being small molecule kinase inhibitors (9). Included among the kinases in the body are 58 receptor tyrosine kinases (RTKs) which are important regulators of various cellular processes and pathways, and many anti-cancer drugs act as receptor tyrosine kinase inhibitors (RTKIs) for the treatment of colorectal cancer liver metastasis (CRLM) (10, 11) and HCC (12). Examples of FDA approved multi-kinase inhibitors include Regorafenib for the treatment of CRLM, and Cabozantinib and Sorafenib for the treatment of HCC by blocking the activity of multiple protein kinases that participate in oncogenesis, tumour angiogenesis, and tumour microenvironment formation (13, 14). Although RTKIs are promising therapeutic agents, the high heterogeneity and mutations in kinases lead to resistance to RTKIs, which is a significant challenge for effective long-term treatment. This emphasizes the need for better understanding of the underlying resistance mechanisms and investigating suitable predictive biomarkers to facilitate personalised therapy (12, 14).

RTKs are cell surface receptors involved in the regulation of important biological pathways and include receptors involved in vascularization (vascular endothelial growth factor receptors; VGFRs), epidermal growth factor receptors (EGFRs), fibroblast growth factor receptors (FGFRs), insulin growth factor receptor (IGFR), platelet-derived growth factor receptors (PGFRs), proto-oncogene c-KIT and others (15). RTKs demonstrate aberrant expression in several cancer types and this is typically associated with poor prognosis (16). The expression levels of kinases vary depending on the stage of cancer (primary or metastatic) (17) and the cancer type (18), indicating the necessity of thorough investigation of different disease stages and types.

Despite the important role of RTKs in cancer, quantitative measurements of these proteins in human cancer are scarce. Data are limited to cell lines using targeted proteomic assays (19), whereas gene expression profiles were previously studied in human gastric cancer cell lines (20) and Ewing sarcoma (21). EGFR and ERBB2 expression was mainly measured by immunohistochemistry methods in primary tumours and CRC metastases (22), whereas ERBB2 and MET expression was measured in colorectal cancer cells in the same population (23). EPHA2 expression and its correlation with cancer progression and metastasis in CRC tissue were also demonstrated (24). In addition, PDGFRB gene expression and its role in epithelial-to-mesenchymal transition (EMT) and metastasis in human CRC cohorts was studied (25). However, immunohistochemistry only provides semi-quantitative protein data and mRNA data does not always correlate with protein levels. LC-MS proteomics is widely used for quantitative measurement of proteins and for identifying important biomarkers in different disease states (26), and therefore, it can contribute to understanding the expression patterns of RTKs in cancer. In a previous pilot study (27), we quantified RTKs in pooled healthy and cancer livers using LC-MS, giving an overall picture of RTK expression for the first time.

The aim of the current study was to quantify human hepatic RTKs and to assess the impact of cancer on their abundance. It was considered important to measure RTK content in individual samples, allowing investigation of covariates that determine the variations of expressions in different individuals in relation to the type of cancer and stage of disease. For this purpose, targeted proteomics was utilized to measure key RTKs in individual membrane fractions from healthy control, non-tumorous (histologically normal) and tumorous matched samples of liver tissue from cancer patients. The cancer cohort in the current study predominantly consisted of CRLM patients, with only two samples in the set from primary hepatic cancer. To the best of our knowledge, this is the first study that quantified RTKs in individual healthy and CRLM subjects and assessed disease impact on abundance. In addition, we were able to clarify the relative distribution of RTKs and highlight significant correlations between various RTKs in each group of samples.

## Materials and methods

### Materials and chemicals

All chemicals and solvents (HPLC grade) were purchased from Sigma-Aldrich (Poole, Dorset, UK) unless otherwise stated. EDTA-free protease inhibitor cocktail and trypsin (sequencing grade) were obtained from Roche Applied Sciences (Mannheim, Germany). Lysyl endopeptidase (Lys-C) was purchased from Wako (Osaka, Japan). The QconCATs were supplied by PolyQuant GmbH (http://www.polyquant.com/) (Germany). Non-naturally occurring peptide (NNOP) standards (light peptides) used for the quantification of QconCATs were purchased from Cambridge Peptides (Cambridge, UK).

### Liver samples

Matched tumorous and non-tumorous (histologically normal) liver tissue from adult cancer patients (n = 18; HCC primary cancer (n = 1), ICC primary cancer (n = 1), CRLM (n = 16)) were obtained from the Manchester University NHS Foundation Trust (MFT) Biobank, Manchester, UK, following hepatectomy. Ethics were covered under the MFT Biobank generic ethics approval (NRES 14/NW/1260 and 19/NW/0644). The cohort comprised 7 female and 11 male participants. The age of the donors ranged between 34 and 85 years, and their body mass index (BMI) varied from 21.6 to 36.3 kg/m^2^. **Table S1** presents demographic and clinical details of the CRLM patients. Healthy human liver microsomal samples from 15 healthy subjects were provided by Pfizer (Groton, CT, USA). These samples were supplied by Vitron (Tucson, AZ, USA) and BD Gentest (San Jose, CA, USA). Ethical approval was covered by the suppliers. Among the 15 donors, 8 were female and 7 were male, and their ages ranged from 18 to 64 years. The BMI of the donors ranged from 19.9 to 37.5 kg/m^2^. **Table S2** presents demographic and clinical details of the healthy subjects.

### Preparation of human liver microsomes

Liver tissue was prepared into microsomes, as previously described (28–30). Briefly, liver tissue was homogenized using a Fisherbrand 150 Handheld Homogenizer (Thermo Fisher Scientific, UK) in homogenization buffer (150 mM KCl, 2 mM EDTA, 50 mM Tris, 1 mM dithiothreitol, and EDTA-free protease inhibitor cocktail, pH 7.4) at 10 ml per gram of liver tissue. Each homogenate sample was centrifuged at 10,000 *g* for 20 min at 4°C using an Optima™ L-100 ultracentrifuge (Beckman Coulter, Fullerton, CA). The pellet (cell debris) was stored at -80°C and the supernatant was further centrifuged at 100,000 *g* for 75 min at 4°C. The cytosol (the supernatant) of each individual sample was stored at -80°C for future use, and the pellet (microsomes) was re-suspended in 1 ml of storage buffer (0.25 M potassium dihydrogen phosphate, 0.25 M dipotassium phosphate, pH 7.25) and stored at -80°C.

### Measurement of total protein content in HLM

The protein content of liver microsomes was measured using bicinchoninic acid (BCA) protein assay (Pierce^®^ Microplate BCA Protein Assay Kit – Reducing Agent Compatible) in triplicate. Absorbance was monitored at 562 nm using a SpectraMax 190 plate reader (Molecular Devices, Sunnyvale, CA), with bovine serum albumin as a calibration standard.

### QconCAT (KinCAT) standard

A novel QconCAT standard, the KinCAT, was used in this study, as described in our previous pilot study (27, 31). It consists of peptides for the quantification of 21 receptor tyrosine kinases (RTKs), concatenated together. To confirm the concentration of the KinCAT, two [Glu^1^]-Fibrinopeptide B analogs (SEGVNNEEGFFSAR and GEGVNNEEGFFSAR) were included in the QconCAT construct. Light (unlabelled) non-naturally occurring peptides (NNOPs) with the same sequences (SEGVNNEEGFFSAR and GEGVNNEEGFFSAR) were used as standards for the quantification of the KinCAT. The target peptides incorporated into the KinCAT belong to the following RTKs: Macrophage colony-stimulating factor 1 receptor CSF1R, Epidermal growth factor receptor EGFR, Ephrin type-A receptor 2 EPH2A, Human epidermal growth factor receptor 2 ERBB2, Fibroblast growth factor receptors FGFR1/2/3, FMS-like tyrosine kinase FLT3, Insulin-like growth factor 1 receptor IGF1R, Insulin receptor INSR, Mast/stem cell growth factor receptor KIT, Hepatocyte growth factor receptor MET, Neurotrophic tyrosine kinase receptor type 2 NTRK2, Platelet-derived growth factor receptors PGFRA/B, Proto-oncogene tyrosine-protein kinase receptor RET, Angiopoietin-1 receptor TIE2, Tyrosine-protein kinase receptor UFO AXL, and Vascular endothelial growth factor receptors VGFR1/2/3. A bacterial ribosome core was added to the KinCAT to facilitate efficient expression and purification (32).

### Digestion and preparation of samples for LC-MS proteomics

A total amount of approximately 70 μg of protein was digested from each microsomal sample and prepared for LC-MS proteomics. 70 μg of protein was digested from most of microsomal samples, with the exception of the following samples: 590 tumour (71 μg digested), HH99 (77 μg digested), 818 histologically normal (77 μg digested), HH101 (83 μg digested), 1492 histologically normal (73 μg digested), and 1492 tumour (76 μg digested). Each sample was mixed with a known amount of isotope-labelled KinCAT; 2.6 µl of 1:5 diluted KinCAT (initial concentration 0.1954 µg/µl). The protein mixtures were solubilized with sodium deoxycholate at a final concentration of 10% (w/v). The mixture was then incubated at room temperature for 10 minutes. Dithiothreitol (DTT) was added at a final concentration of 0.1 M to reduce disulphide bonds and the mixture was incubated at 56°C for 30 minutes. For protein digestion, filter-aided sample preparation (FASP) was used, as previously described (33–35) with minor modifications. Amicon Ultra 0.5 ml centrifugal filters at 10 kDa molecular weight cut-off (Merck Millipore, Nottingham, U.K.) were conditioned with 200 μl of 0.1 M Tris, pH 8.5, followed by centrifugation in a microfuge at 14,000 rpm at room temperature for 10 minutes, this step was repeated twice. The reduced protein samples were added to the filters and centrifuged at 13,000 rpm for 20 minutes. A volume of 200 μl of 8 M urea in 0.1 M Tris-HCL, pH 8.5, was added for buffer exchange, followed by centrifugation at 14,000 rpm for 20 minutes at room temperature (twice). The samples were subsequently alkylated with 100 µl of 50 mM iodoacetamide (IAA) in the dark for 30 minutes at room temperature, followed by centrifugation at 14,000 rpm for 10 minutes. Two washes were carried out with 8 M Urea in 0.1 M Tris, pH 8.5, and centrifugation at 14,000 rpm for 20 minutes at room temperature. Buffer exchange was carried out using two washes of 1 M urea in 50 mM ammonium bicarbonate, pH 8.0, followed by centrifugation at 13,000 rpm for 20 minutes. The proteins were in the filter up to this step and the washes were discarded. 80 μl of 1 M urea in 50 mM ammonium bicarbonate solution pH 8.0 was added to each filter unit, and lysyl endopeptidase was applied to each sample (enzyme:protein ratio of 1:50) for two hours at 30°C (twice). Trypsin was then added (enzyme:protein ratio 1:25) for 12 hours at 37°C, and the trypsin proteolysis step was repeated for an extra four hours. The peptides were recovered by centrifugation at 14,000 rpm for 20 minutes. A volume of 100 μl of 0.5 M sodium chloride was added to the filters, followed by centrifugation at 14,000 rpm for 20 minutes. This step was repeated using 50 μl of 0.5 M sodium chloride. Each collected sample was then split into two equal volumes, and each was mixed with sample buffer (3 parts sample:1 part sample buffer, 2% v/v trifluoroacetic acid in 20% v/v acetonitrile in water). Each sample was then desalted using a C18 column (Nest group, USA). Finally, the peptide samples were lyophilized using a vacuum concentrator and stored at −80°C until LC-MS/MS analysis. Reconstitution buffer (3% acetonitrile-0.1% formic acid) and unlabelled peptides [SEGVNNEEGFFSAR (0.176 pmol) and GEGVNNEEGFFSAR (0.176 pmol)] were added at a final volume of 60 µl before the LC-MS/MS.

### Liquid chromatography and tandem mass spectrometry

Digested samples were analysed by LC-MS/MS using an UltiMate^®^ 3000 Rapid Separation LC (RSLC, Dionex Corporation, Sunnyvale, CA) coupled to a Q Exactive HF (Thermo Fisher Scientific, Waltham, MA) mass spectrometer. Mobile phase A was 0.1% formic acid in water and mobile phase B was 0.1% formic acid in acetonitrile and the analytical column was a 75 mm x 250 μm, i.d. 1.7 μM, CSH C18 column (Waters, UK). 1 µl of the sample was transferred to a 5 µl loop and loaded onto the column at a flow of 300 nl/min for 5 minutes at 5% B. The loop was then taken out of line and the flow was reduced from 300 nl/min to 200 nl/min in 0.5 minutes. Peptides were separated using a gradient from 5% to 18% B in 63.5 minutes, then from 18% to 27% B in 8 minutes and finally from 27% B to 60% B in 1 minute. The column was washed at 60% B for 3 minutes before re-equilibration to 5% B in 1 minute. At 85 minutes, the flow was increased to 300 nl/min until the end of the run at 90 minutes. Mass spectrometry data was acquired in a data dependent manner for 90 minutes in positive ionization mode. Peptides were selected for fragmentation automatically by data dependant acquisition on the basis of the top 12 peptide ions with m/z between 300 to 1750 Th and a charge state of 2, 3 or 4 with dynamic exclusion set at 15 sec. MS1 resolution was set at 120,000 with an AGC target of 3e6 and a maximum fill time set at 20 ms. MS2 resolution was set to 30,000, with an AGC target of 2e5, a maximum fill time of 45 ms, isolation window of 1.3 Th and a collision energy of 28 eV.

### Analysis and annotation of proteomic data

Proteomic data were processed using MaxQuant 1.6.7.0 (Max Planck Institute, Martinsried, Germany) and searched against a customized database, comprising human UniprotKB database (74,788 sequences) and QconCAT sequences. For targeted analysis, light-to-heavy MS intensity ratios were used with QconCAT concentration to calculate protein amounts based on accurate mass and retention time measurements for each peptide (34, 36). Peptides selected for quantification of RTKs are presented in **Tables S4-S6**. The mass spectrometry proteomic data have been deposited to the ProteomeXchange Consortium *via* the PRIDE (37) partner repository with the data set identifier: PXD038776.

### Statistical data analysis

Statistical data analysis was performed using GraphPad Prism 8.1.2 (La Jolla, California USA). Non-parametric statistics were used because the data did not follow normal distribution. Differences in absolute abundances between healthy and histologically normal livers, between healthy and tumorous livers, and between histologically normal and tumorous livers were assessed using the Mann−Whitney U-test. Histologically normal and tumour samples are matched but abundance data were not always available for all targets in each sample pair. For targets where data were available in matched samples for the same donors, differences in absolute abundances were assessed using the Wilcoxon test. For the assessment of correlations among RTKs and between RTKs and other proteins, Spearman correlation and linear regression analysis were used. The probability cut-off for statistical significance was set at p < 0.05.

## Results

The main objective of this study was to measure the abundance of RTKs that play important biological roles in CRLM and could be used as input to systems biology models of cancer and ultimately as biomarkers of disease progression that helps improve drug therapy. For the first time, the absolute abundance of 21 pharmacologically important RTKs was measured using LC-MS/MS proteomics with a QconCAT standard (KinCAT), which we previously designed and tested (27, 31). RTK abundance was expressed as pmol of protein per mg of microsomal protein and the abundance levels of each RTK target in liver tissues taken from healthy subjects were compared with those from matched tumorous and non-tumorous (histologically normal) liver tissue from cancer patients.

### Differential abundance of RTKs in HLMs from healthy subjects, and paired non-tumorous (histologically normal) and tumorous samples from cancer patients

**Figure 1:**
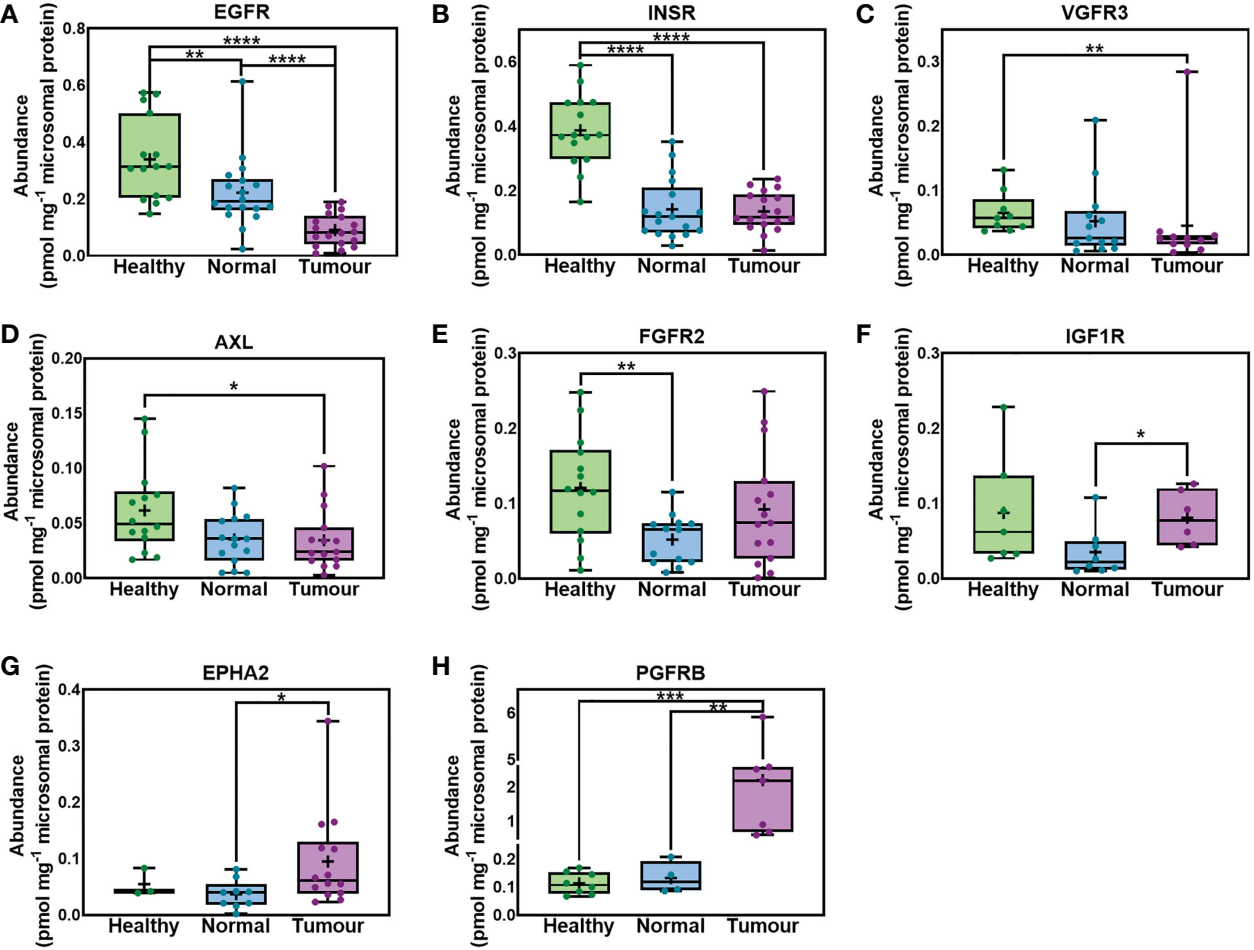
Absolute abundance of receptor tyrosine kinases (RTKs) with different levels of expression in samples taken from healthy individuals, non-tumorous liver (histologically normal) tissue and matching tumorous livers from cancer patients. EGFR **(A)**, INSR **(B)**, VGFR3 **(C)**, AXL **(D)**, FGFR2 **(E)**, IGF1R **(F)**, EPHA2 **(G)** and PGFRB **(H)** are depicted. The whiskers are the minimum and maximum values, the boxes represent the 25th and 75th percentiles, the lines show the medians, and the + signs are the means. The dots represent individual values. Mann−Whitney test was used to assess differences between pairs of the sets of samples for each protein; *p < 0.05, ** p < 0.01, ***p < 0.001, **** p < 0.0001.

**Figure 1** shows that several RTKs were expressed at different levels among healthy, non-tumorous (histologically normal) and tumorous liver tissues from cancer patients. EGFR was significantly lower in non-tumorous (histologically normal) tissue and tumours compared with healthy controls, and in tumours vs matching non-tumorous liver tissue from the same patients. INSR was expressed at significantly lower levels in non-tumorous (histologically normal) and tumorous tissue relative to healthy controls. VGFR3 and AXL were significantly downregulated in tumours compared with healthy livers from healthy individuals. Comparing the abundance of FGFR2 in livers from healthy subjects versus non-tumorous (histologically normal) tissue taken from cancer patients and tumorous samples, we found significantly lower values in non-tumorous tissue but no statistically significant change in tumorous samples. On the other hand, IGF1R and EPHA2 significantly increased in tumorous samples compared with matching non-tumorous (histologically normal) samples. Lastly, PGFRB was significantly upregulated in tumorous compared with matching non-tumorous (histologically normal) tissue and samples taken from healthy individuals.

**Figure 2:**
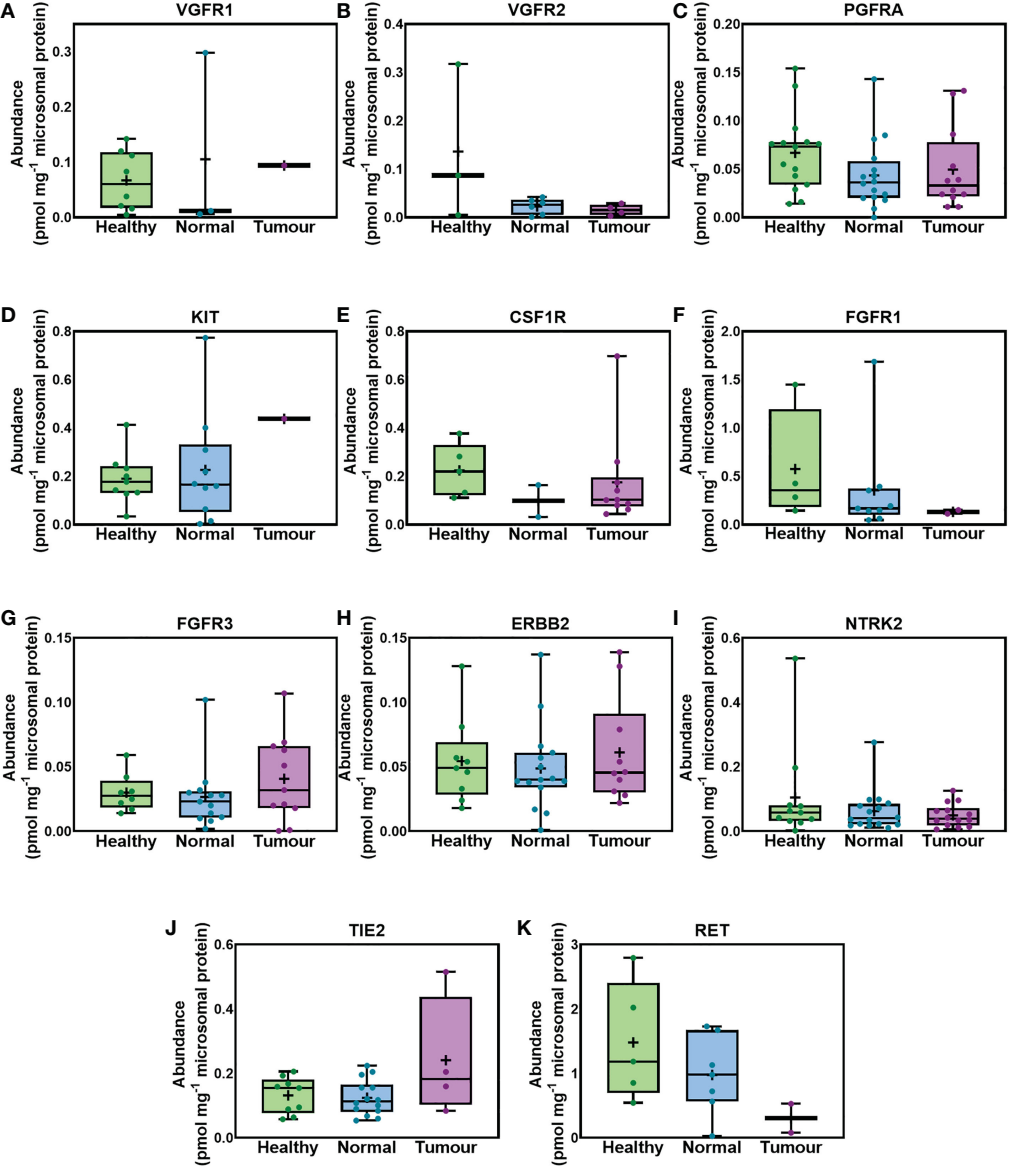
Absolute abundance of RTKs similarly expressed in healthy, histologically normal and tumorous tissue. VGFR1 **(A)**, VGFR2 **(B)**, PGFRA **(C)**, KIT **(D)**, CSF1R **(E)**, FGFR1 **(F)**, FGFR3 **(G)**, ERBB2 **(H)**, NTRK2 **(I)**, TIE2 **(J)** and RET **(K)** are depicted. The whiskers show the minimum and maximum values, the boxes represent the 25th and 75th percentiles, the lines show medians, and the + signs are the means. The dots represent individual values. Mann−Whitney test was used to assess differences between pairs of the sample sets. No significant differences were observed (p > 0.05).

**Figure 2**, for first time, provides abundance values for 11 RTKs in liver samples taken from healthy individuals, and matching tissue of liver from cancer patients representing the non-tumorous (histologically normal) and tumorous regions. As indicated in the figure there were no statistically significant differences among the 3 sets. Statistical analysis was performed in cases where RTKs were detected in more than 3 samples per dataset (healthy, non-tumorous and tumorous livers). No statistical difference was observed in the abundance of VGFR2, PGFRA, FGFR3, ERBB2, NTRK2 and TIE2 among the healthy, non-tumorous (histologically normal) and tumorous livers from cancer patients. VGFR1, KIT, FGFR1 and RET were expressed at similar levels in healthy and non-tumorous (histologically normal) livers from cancer patients. No comparison was possible between tumorous and non-tumorous or healthy livers for VGFR1, KIT, FGR1 and RET because these targets were only quantifiable in 1 (VGFR1, KIT) or 2 tumorous samples (FGFR1, RET). Similar abundance of CSF1R was observed between healthy and tumorous livers from cancer patients. CSF1R was only quantified in one non-tumorous (histologically normal), and thus no comparison between non-tumorous and healthy or tumorous livers was possible. MET was only quantifiable in two tumour samples, while FLT3 was only quantifiable in 1 tumour sample. Some RTKs are low abundance proteins and therefore, they were not quantifiable in all the samples.

**Figure S1** depicts the individual RTK abundance values that were measured in non-tumorous (histologically normal) and matched tumorous samples for each individual (paired). These proteins were quantified in three or more than three pairs of samples. **Figure S2** shows the lower limit of quantification (LLOQ), the precision and accuracy, based on replicate measurements in a pool of healthy samples. In addition to the validation of the data using the precision and accuracy based on replicates, the data were also validated based on our previous study (30). That study used the same dataset and quantified drug metabolising enzymes and transporters and showed consistency with the literature data for healthy livers. Therefore, these findings validate the experimental data. The remaining amounts for each sample are minimal and therefore, assays such as western blot or immunohistochemistry could not be performed to additionally validate the data in this way too. However, in the past we compared LC-MS and immunoblotting by quantifying the abundance of CYP3A4 and CYP3A5 in human liver microsomes, and we confirmed consistency between the two methods (38). Therefore, we expect consistency for RTKs and other proteins.

**Tables S3** provides the abundance levels of all the measured RTKs in healthy, non-tumorous (histologically normal) and tumour tissues expressed as median, mean, standard deviation of the mean (SD), coefficient of variation (CV), and minimum to maximum range. Individual absolute values of the abundance of RTKs in healthy, non-tumorous (histologically normal) and tumour liver samples are provided in **Tables S4-S6**.

**Table 1** summarizes the RTKs that are overexpressed, suppressed or do not change in the liver of cancer patients compared with healthy controls. The *p* values are presented in cases of overexpression or suppression.

**Table 1:** Overexpression, suppression, and no changes of RTKs in cancer.

Overexpression of RTKs in Cancer	
Healthy Liver Tissue = Non-Tumorous Tissue from Cancer Patients, < Tumorous Tissue from Cancer Patients	
PGFRB (H: 0.11 ± 0.04, N: 0.13 ± 0.06, T: 2.2 ± 1.85), (H < T, *p* < 0.001; N < T, *p* < 0.01)
Healthy Liver Tissue = Non-Tumorous/Tumorous Tissue from Cancer Patients; Non-Tumorous Tissue from Cancer Patients < Tumorous Tissue from Cancer Patients	
IGF1R (N: 0.04 ± 0.03, T: 0.08 ± 0.04, *p* < 0.05) EPHA2 (N: 0.04 ± 0.03, T: 0.09 ± 0.09, *p* < 0.05)
Suppression of RTKs in Cancer	
Healthy Liver Tissue > Non-Tumorous Tissue from Cancer Patients > Tumorous Tissue from Cancer Patients	
EGFR (H: 0.34 ± 0.15, N: 0.22 ± 0.12, T: 0.09 ± 0.06), (H > N, *p* < 0.01; H > T, *p* < 0.0001; N > T, *p* < 0.0001)
Healthy Liver Tissue > Non-Tumorous Tissue from Cancer Patients; Healthy Liver Tissue > Tumorous Tissue from Cancer Patients	
INSR (H: 0.39 ± 0.11, N: 0.14 ± 0.09, T: 0.13 ± 0.06), (H > N, *p* < 0.0001; H > T, *p* < 0.0001)
Healthy Liver Tissue > Non-Tumorous Tissue from Cancer Patients; Healthy Liver Tissue = Tumorous Tissue from Cancer Patients	
FGFR2 (H: 0.12 ± 0.07, N: 0.05 ± 0.03, *p* < 0.01)
Healthy Liver Tissue = Non-Tumorous Tissue from Cancer Patients; Healthy Liver Tissue > Tumorous Tissue from Cancer Patients	
VGFR3 (H: 0.07 ± 0.03, T: 0.05 ± 0.08, *p* < 0.01) AXL (H: 0.03 ± 0.03, T: 0.06 ± 0.04, *p* < 0.05)
No Changes of RTKs in Cancer	
Healthy Liver Tissue = Non-Tumorous Tissue from Cancer Patients = Tumour Tissue form Cancer Patients	
VGFR1 (H: 0.07 ± 0.05, N: 0.11 ± 0.17)
VGFR2 (H: 0.14 ± 0.16, N: 0.02 ± 0.02, T: 0.02 ± 0.01)
PGFRA (H: 0.07 ± 0.04, N: 0.04 ± 0.04, T: 0.05 ± 0.04)
KIT (H: 0.19 ± 0.11, N: 0.23 ± 0.23)
CSF1R (H: 0.22 ± 0.11, T: 0.18 ± 0.19)
FGFR1 (H: 0.58 ± 0.59, N: 0.36 ± 0.51)
FGFR3 (H: 0.03 ± 0.01, N: 0.03 ± 0.03, T: 0.04 ± 0.03)
ERBB2 (H: 0.05 ± 0.03, N: 0.05 ± 0.03, T: 0.06 ± 0.04)
NTRK2 (H: 0.1 ± 0.15, N: 0.06 ± 0.06, T: 0.05 ± 0.04)
TIE2 (H: 0.13 ± 0.06, N: 0.12 ± 0.06, T: 0.24 ± 0.19)
RET (H: 1.48 ± 0.92, N: 0.98 ± 0.61)

H: abundance of RTKs in healthy liver tissue, N: abundance of RTKs in non-tumorous liver tissue from cancer patients, T: abundance of RTKs in tumorous liver tissue from cancer patients. The abundance is expressed in pmol of protein per mg of microsomal protein. Mann−Whitney test was used to assess differences between pairs of the sets of samples for each protein.

### Correlations in liver RTK abundance profiles

**Figure 3:**
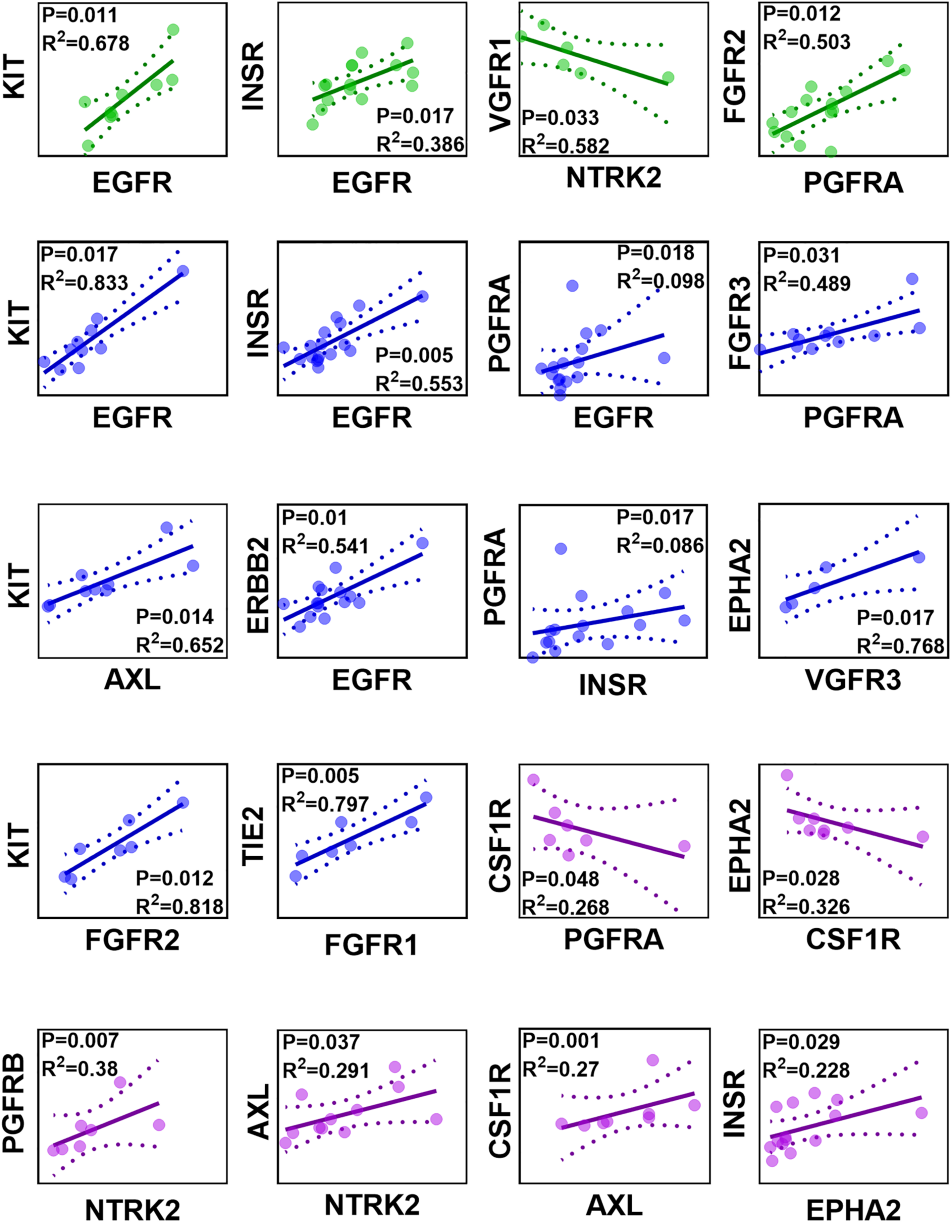
Correlation matrix of protein abundance of RTKs in healthy (green), non-tumorous (histologically normal) (blue) and tumorous (purple) samples. Abundance values are expressed in pmol of protein per mg of microsomal protein. Strong and significant correlations (Spearman rank-order correlation coefficient Rs > 0.60, *p* < 0.05) and moderate and significant correlations (Rs > 0.50, *p* < 0.05) are presented. The solid lines are the regression lines. The dotted curves represent the upper and lower confidence interval (CI) limits; 95% confidence interval of the slope. The sample observations are represented by circles.

The correlation of protein abundance between different RTKs was assessed in healthy, non-tumorous (histologically normal) and tumorous samples, and only significant correlations (*p* < 0.05) are presented in **Figure 3**. Strong (Rs > 0.60, with very limited scatter, R^2^ > 0.30) and moderate (Rs > 0.50 and R^2^ > 0.25) correlations were considered.

INSR and EGFR were found to have a strong, significant, and positive correlation in healthy livers, as were correlations between KIT and EGFR and between FGFR2 and PGFRA. By contrast, VGFR1 and NTRK2 had a significant, negative correlation. In non-tumorous (histologically normal) livers, TIE2 and FGFR1, INSR and EGFR, ERBB2 and EGFR, KIT and EGFR, KIT and AXL, EPHA2 and VGFR3, FGFR3 and PGFRA, and KIT and FGFR2 had strong, significant, and positive correlations. Although significant (*p* < 0.05) and moderate (Rs > 0.50) correlations were observed between PGFRA and EGFR, and PGFRA and INSR, the scatter was very high (R^2^ < 0.1). In tumours, a strong, significant and positive correlation was recorded between PGFRB and NTRK2. Strong, significant and positive correlations (Rs > 0.60, R^2^ > 0.25) were observed between AXL and NTRK2, and CSF1R and AXL. The correlation between INSR and EPHA2 was moderate, significant and positive (Rs > 0.50, R^2^ < 0.25). Lastly, a strong, significant, and negative correlation was observed between EPHA2 and CSF1R, and a moderate, significant, and negative correlation was recorded between CSF1R and PGFRA.

### Relative abundance of RTKs in matching non-tumorous and tumorous liver tissue from cancer patients compared with tissues taken from healthy individuals

**Figure 4:**
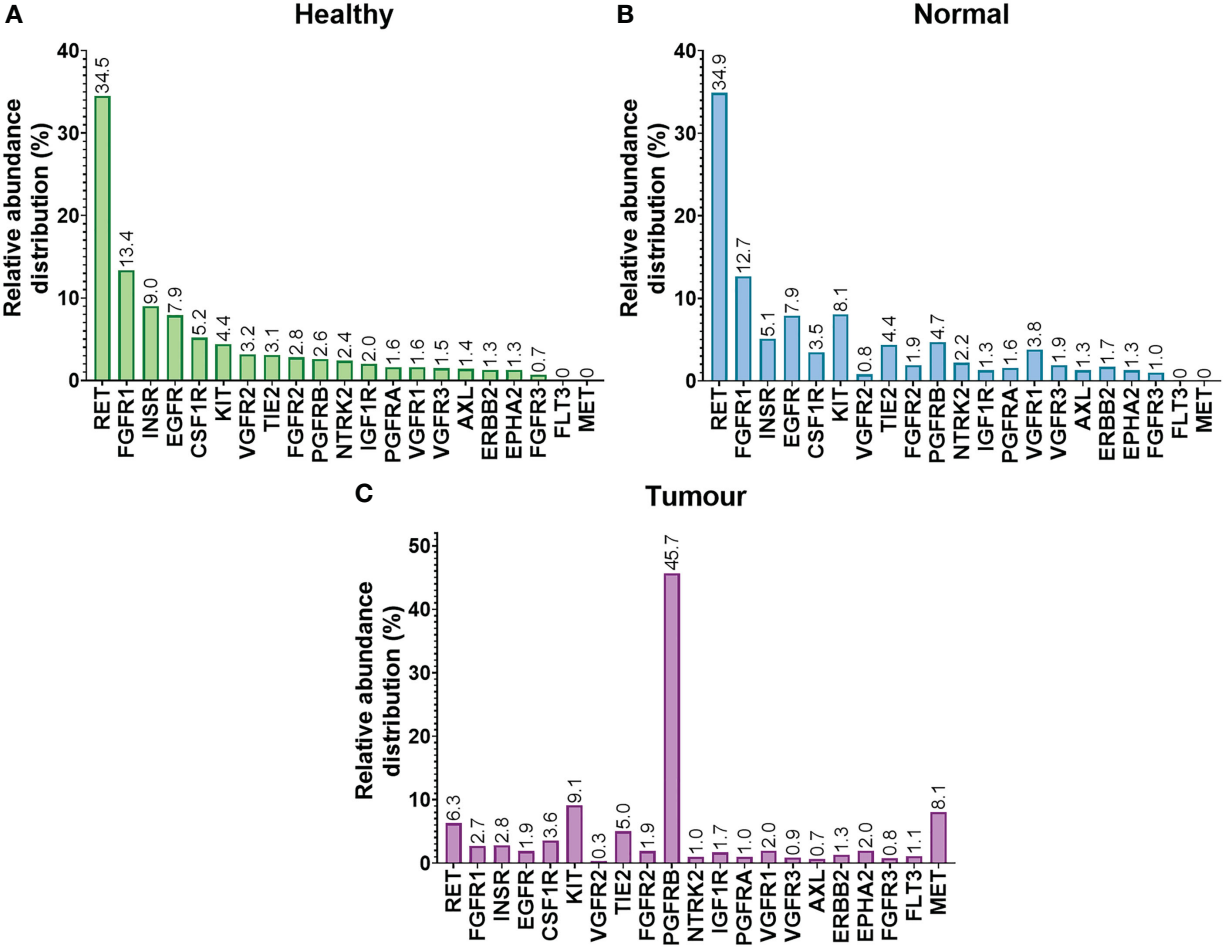
Relative abundance distribution (%) of RTKs in healthy **(A)**, non-tumorous (histologically normal) **(B)** and tumorous **(C)** liver sets.

Relative abundance of RTKs in liver tissues taken from healthy controls are shown in **Figure 4A**. Corresponding relative abundances in non-tumorous and tumorous tissues from cancer patients are shown in **Figures 4B, C**, respectively. The relative abundance of RTKs was broadly similar in samples from healthy individuals when compared to non-tumorous tissue taken from liver of cancer patients, with RET being the most abundant. The second most abundant RTK in these samples was FGFR1 (13.4% and 12.7%, respectively). The least abundant RTKs in healthy livers were FGFR3 (0.7%), EPHA2 and ERBB2 (1.3%), while in non-tumorous (histologically normal) tissue these were VGFR2 (0.8%) and FGFR3 (1%).

The relative proportion of various RTKs differed in tumours. The most abundant RTK was PGFRB, representing almost half of the quantified RTKs (45.7%), followed by KIT (9.1%), MET (8.1%), and RET (6.3%). The least abundant RTKs were VGFR2 (0.3%) and AXL (0.7%).

### The effect of demographics on the abundance levels of RTKs

**Figure 5:**
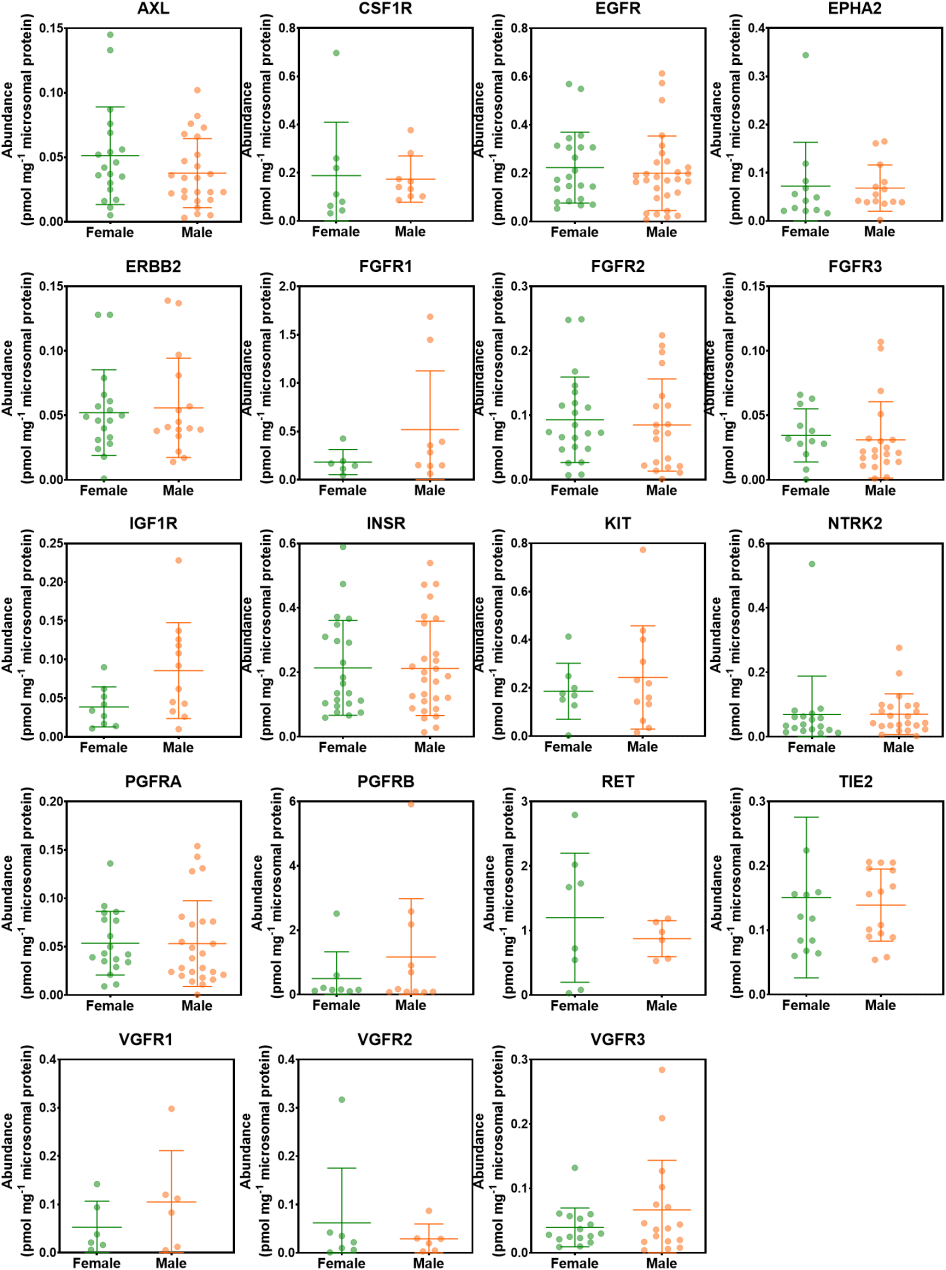
Abundance levels of RTKs in female and male donors. Mann−Whitney test was used to assess differences between female and male donors. No significant differences were observed (p > 0.05).

The effect of sex on the abundance of RTKs was assessed. **Figure 5** shows the abundances of RTKs in healthy, non-tumorous (histologically normal) and tumorous livers in female donors compared with their male counterparts. Data in healthy, non-tumorous and tumorous livers were grouped together, and there were no statistically significant differences between abundance patterns in females and males (p > 0.05). The analysis was also done for each of the liver groups (healthy, non-tumour, tumour), and again, no sex differences were observed for all measured RTKs.

**Figure 6:**
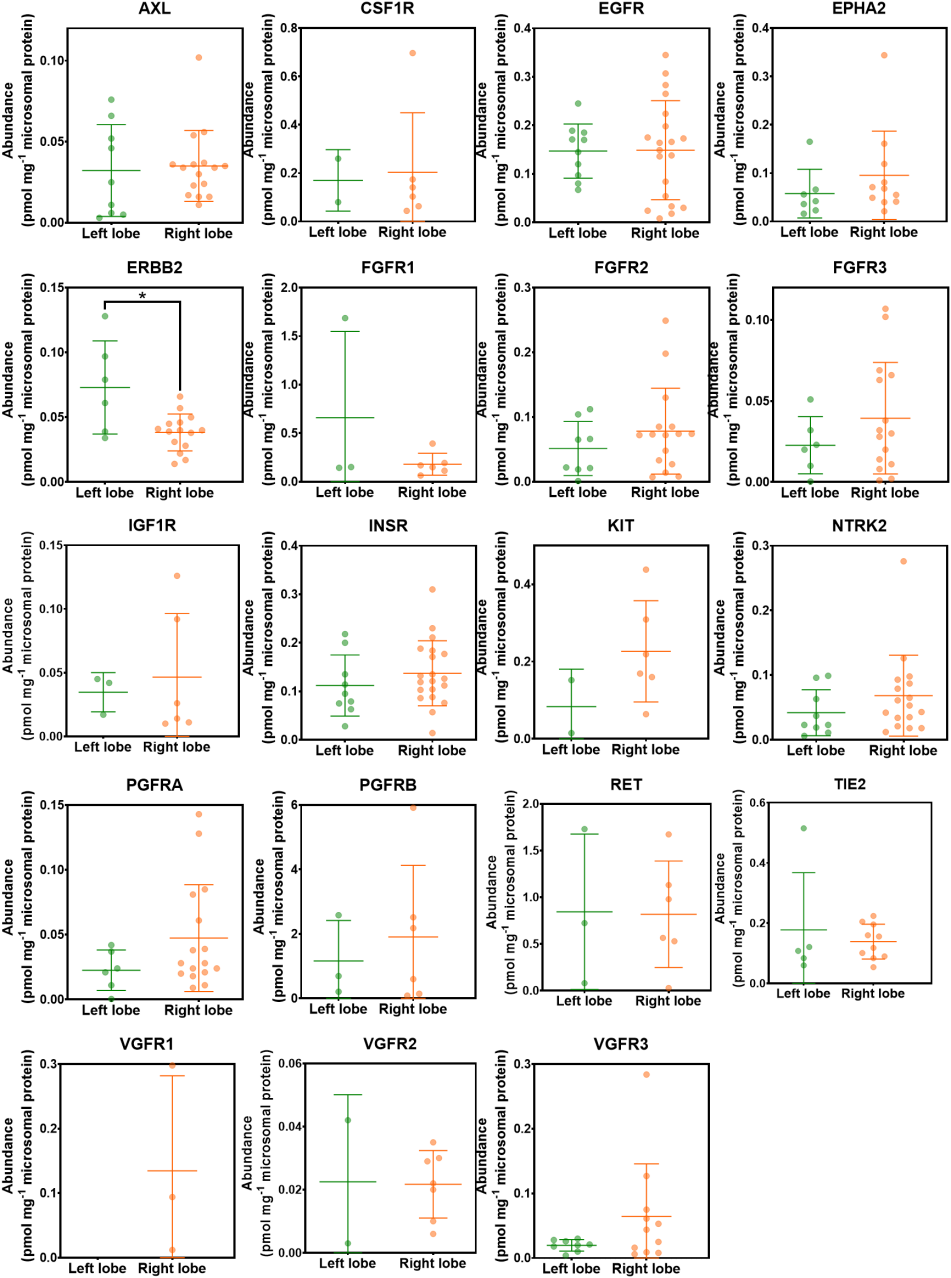
Abundance levels of RTKs in left and right hepatic lobes. Mann−Whitney test was used to assess differences between left and right hepatic lobes for each protein; *p < 0.05.

The effect of liver lobe on the abundance of RTKs was investigated. **Figure 6** categorises the liver samples into two groups; right and left hepatic lobes. The non-tumorous (histologically normal) and tumorous livers were analysed together as one group. The healthy controls were not included in this analysis because liver lobe information was not available. ERBB2 was significantly higher in the left liver lobe compared with the right liver lobe. However, there was no statistically significant difference in abundance of the other RTKs between the right and left lobes (p > 0.05). The analysis was repeated for each of the liver groups (non-tumour, tumour) separately, and no statistical difference was observed between the two lobes.

The effect of the age on the abundance of RTKs was assessed in all liver samples; there were significant and negative correlations between donor age and abundance of FGFR1 (p < 0.05), VGFR3 (p < 0.05), EGFR (p < 0.01), INSR (p < 0.001) and CSF1R (p < 0.001). However, when healthy controls were studied alone, we observed positive and significant correlations (p < 0.05) for FGFR2, FGFR3 and TIE2. In non-tumorous (histologically normal) livers, there was positive correlation for RET (p < 0.05), and negative correlations for EGFR (p < 0.05), ERBB2 (p < 0.01), and TIE2 (p < 0.01). In tumours, no significant correlations were found.

Another explored parameter was BMI. When the samples were assessed collectively, there was no correlation between BMI and abundance of RTKs. The same trend was observed for the cancer set. However, there was a negative correlation between the abundance of PGFRB and BMI (p < 0.05) in healthy livers and a negative correlation for RET (p < 0.05) in non-tumorous (histologically normal) livers.

### Correlations of the abundance between RTKs and other proteins

Drug metabolising enzymes (DMEs), such as cytochrome P450 (CYP) and UDP-glucuronosyltransferase (UGT) enzymes, and drug transporters, such as ATP-binding cassette (ABC) transporters and solute carriers (SLC), govern metabolism and disposition of drugs, including small molecule TKIs. We previously (27, 30, 39) showed that DMEs and transporters are perturbed in CRLM, thus affecting the pharmacokinetics (PK) of several drugs. We also showed previously (27) and in the current report that RTKs are also affected in CRLM and this could lead to different pharmacodynamic (PD) profiles in different patients. Therefore, knowing the impact of cancer on the abundance of DMEs, transporters and RTKs could facilitate precision dosing in CRLM patients. For this purpose, we investigated any potential correlations between RTKs and CYPs, UGTs, ABC transporters, and SLCs.

**Table S7** provides details on correlations (Spearman rank-order correlation coefficient, Rs) that are statistically significant (*p* < 0.05). All liver samples (healthy, non-tumorous and tumorous) were assessed together.

**Figure 7:**
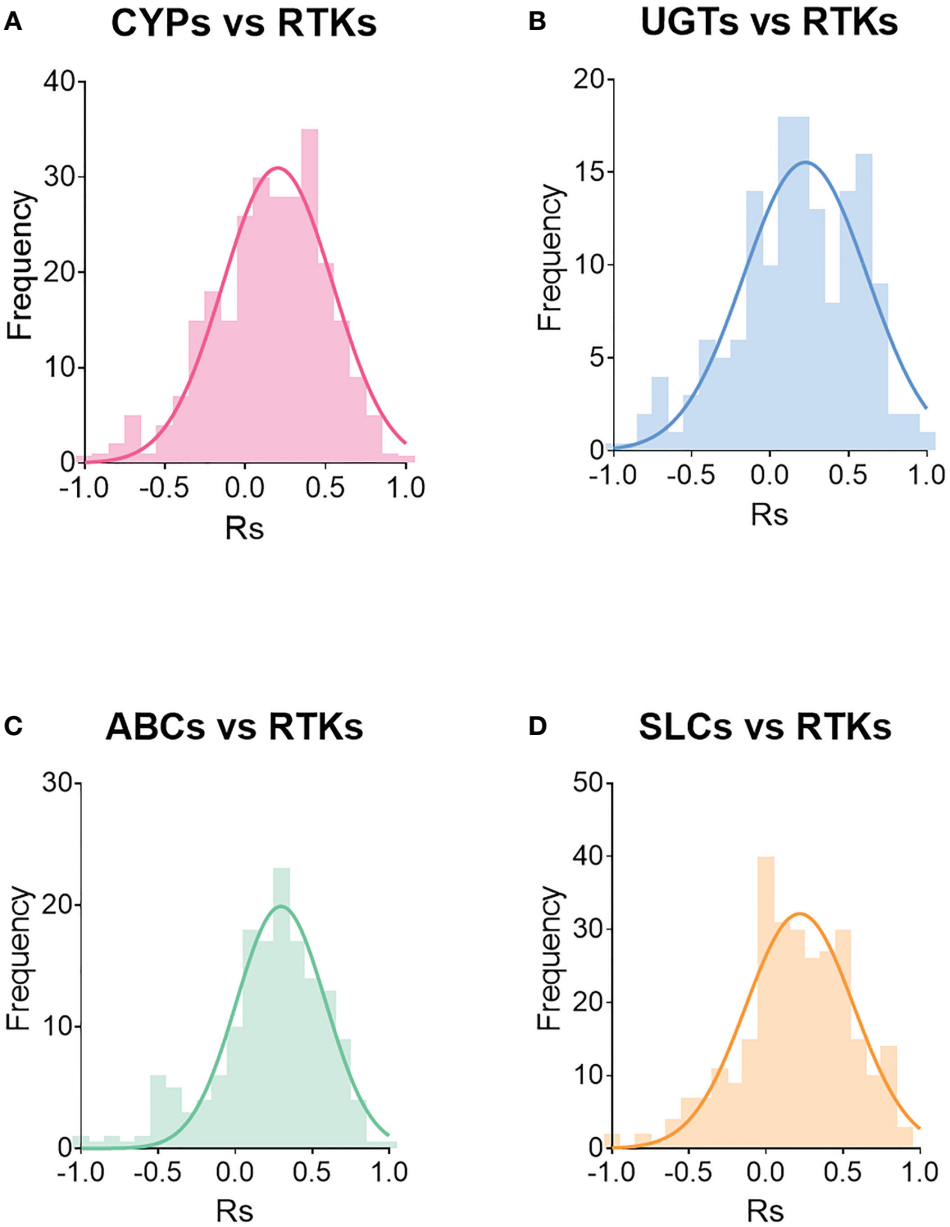
Distribution of correlations between RTKs and CYPs **(A)**, UGTs **(B)**, ABC transporters **(C)** and SLCs **(D)**. Rs is Spearman’s correlation coefficient.

**Figure 7** depicts the distribution of the frequency of the correlations, while **Figure S3** shows the distribution of statistically significant (*p* < 0.05) correlations. Most of the significant correlations are moderate to strong, with Rs > 0.5 or Rs < -0.5. For example, 76% of the significant correlations between CYPs and RTKs had Rs values ranging from -0.9 to -0.5 or from 0.5 to 0.9. Notably, 91% of the significant correlations between UGTs and RTKs had Rs ranging from -0.8 to -0.5 and from 0.5 to 0.9. For transporters, 69% of significant correlations between ABC transporters and RTKs and 80% between SLCs and RTKs were moderate (Rs < -0.5 and Rs between 0.5 and 0.8 for ABC transporters, and Rs ranging from -0.9 to -0.5 and from 0.5 to 0.9 for SLCs).

## Discussion

The current study defines, for the first time, quantitative protein abundance profiles of receptor tyrosine kinases in healthy, non-tumorous (histologically normal) and liver metastasis tissues. RTKs are key targets for the treatment of cancer, and this raises the need for better understanding of disease-related alterations that occur in their abundance. 15 healthy samples and 18 pairs of tumorous and peri-carcinomatous tissue were examined for the abundance of 21 RTKs using LC-MS/MS proteomics, allowing RTKs relative distribution across the three sets and correlations among different RTKs to be explored.

Interestingly, we showed increased abundance of EPHA2 and much more massive increase of PGFRB in tumours compared with non-tumorous (histologically normal) tissue, consistent with our previous pilot study (27). EPHA2 regulates tumour initiation, vascularization, tumour progression and metastasis, and immunohistochemistry in colorectal tumours indicated significantly higher expression of EPHA2 compared with matched non-tumorous (histologically normal) tissue (24, 40). A potential mechanism behind the increased abundance of EPHA2 in tumour could be the regulation by E-cadherin that regulates the function and expression of EPHA2 (24). Higher PGFRB expression was also in agreement with the literature, as PGFRB overexpression is associated with angiogenesis, invasion, metastasis, and poor survival in CRC and is a biomarker for diagnosis and treatment (25, 41). It has been shown that PGFRB signalling in mesenchymal-like tumour cells is responsible for liver metastasis in CRC and its expression is associated with the activation of platelets, transforming growth factor beta (TGFB) signalling, and epithelial-to-mesenchymal transition, which takes place during metastasis (25). All these findings suggest that EPHA2 and especially PGFRB may be very promising drug targets for the treatment of CRLM patients.

Our data suggest significantly low abundance of EGFR in non-tumorous (histologically normal) and lower abundance in tumorous compared with healthy livers, and this may indicate high risk of developing cancer in individuals with low amounts of EGFR. Previous studies support the role of EGFR in the regulation of cell proliferation, differentiation, and migration (42), and its over-expression has been linked with relatively poor prognosis for survival for CRC patients (43). However, a decrease was apparent in our tumorous livers when comparing with matched non-tumorous samples from same donors, in line with immunohistochemistry data indicating that EGFR is lost in several metastasising primary colorectal cancer tumours (22). A potential mechanism behind the downregulation of EGFR may be the hyper-methylation of the promoter that may be associated with resistance to anti-EGFR treatment in CRC (44). Additionally, it has been shown (in breast cancer) that systemic dissemination may lead to internalization and downregulation of EGFR in a metastatic environment leading to resistance to EGFR inhibitors (45). CRLM is a case of metastasis and similar mechanisms may occur, but these need further investigation. Overall, all these imply that EGFR may not be a suitable drug target for CRLM patients.

In addition, INSR and IGF1R are important for energy metabolism, cell growth, and cancer progression (46), and to our knowledge, no quantitative data have been published for INSR. Literature data from immunostaining experiments on IGF1R are contradictory (47): increased risk of liver metastasis in CRC correlated with low expression of IGF1R (48), strong positive correlation of IGF1R higher grade (49), and no correlation with tumour grade and metastasis (50). In our study, for the first time, INSR was expressed at lower abundance in non-tumorous (histologically normal) tissue and tumours compared with healthy samples. It has been shown that when the abundance of INSR is increased (in breast cancer), there is poor survival for the patients (51). By contrast, IGF1R was significantly higher in tumours. IGF1R can contribute to chemotherapeutic drug resistance through the mechanisms of cell proliferation, inhibition of apoptosis, interplay with the extracellular matrix formation and ABC transporters (52).

Immunohistochemistry data showed that VGFR3 is associated with vascularization and hepatic metastasis in CRC patients (53), while several studies have shown that expression of AXL was elevated in advanced CRC, which may be associated with poor survival (54). However, in our study, VGFR3 and AXL were significantly decreased in tumour compared with healthy controls. Several mechanisms may be responsible for the downregulation of AXL, involving promoter hypermethylation, presence of certain miRNAs and problematic protein folding when heat shock protein 90 (HSP90) chaperone is inhibited (55). VEGF-C binds to its receptor VGFR3 and when it is downregulated, the tumour growth and metastasis are inhibited (56). Previous studies have demonstrated the importance of FGFR2 for cell migration, invasion, growth and cancer progression in CRC (57), with gene amplification being reported in primary CRC (58), and expression being associated with poor survival (59). In our data, the abundance of FGFR2 was significantly decreased in non-tumorous (histologically normal) relative to healthy tissue.

Several of the studies discussed above reported an overexpression of RTKs in primary CRC. We, however, measured RTKs in metastatic tumours, and there is some evidence that these tumours lose the expression of RTKs as described in an immunohistochemistry study showing that EGFR is lost in metastatic CRC cancer (22). This indicates that the expression of RTKs may not follow the same patterns in primary and secondary tumours so these should be considered as different diseases with different therapeutic needs. The therapeutic approach may need to be repurposed in these patients fulfilling the inter-individual needs. Further, there is considerable variation among the diseased population, suggesting very different therapeutic requirements by different patients. This could explain the poor response to kinase inhibitors in CRLM patients. We measured absolute abundance of RTKs for the first time using a mass spectrometry-based proteomic approach. The technique is very sensitive and selective. By contrast, immunoquantification provides semi-quantitative data and mRNA measurements in tissue do not always reflect protein abundance (60). For future studies, we advocate comparing RTKs in matched primary and secondary tumours to assess whether their expression is lost in metastatic tumours.

To our knowledge, this is the first study to assess the relative distributions of RTKs in healthy, and matched non-tumorous and tumorous livers samples from cancer patients. Proto-oncogene tyrosine-protein kinase receptor RET was the most abundant RTK (more than one third of the quantified RTKs) in healthy and non-tumorous (histologically normal) tissue. This is not surprising as RET is a tumour suppressor gene. It may promote colon cancer when it is hyper-methylated and inactivated but it leads to apoptosis when restored (61). High expression of RET in healthy and non-tumorous (histologically normal) tissues is indicative of lack of inactivation of the gene and thus, the absence of tumours. On the contrary, the relative distribution differs in tumour, with PGFRB being the most abundant RTK (45.7%). This is in line with literature data discussed above describing PGFRB as a diagnostic and therapeutic marker for CRLM. Not only the distribution of RET and PGFRB change in cancer but also the distribution of several other RTKs, affecting the regulation of cancer pathways. Therefore, the kinase profile is different in CRLM patients and should be carefully considered to decide which kinase inhibitor would be ideal for these patients.

RTKs are regulators of various complex pathways in cells and should be investigated in relation to their pathways rather than as individual proteins. Therefore, it is important to elucidate their relationship in the cells and find any possible correlations between them, which may identify important diagnosis and treatment markers. Interestingly, we found several positive and negative correlations among RTKs in healthy, non-tumorous (histologically normal) and tumorous livers. These correlations are novel, and the data highlight that there is a level of interplay among RTKs, which may be important for suggesting a panel of diagnostic markers or a set of proteins that should be targeted by multi-kinase inhibitors for appropriate treatment. The number of samples in this study may however be a limiting factor. Hopefully, in the future we will be able to provide more details in the molecular mechanisms and pathways where RTKs are involved using label-free proteomics data from the same samples (Vasilogianni et al., manuscript in preparation).

While RTKs are important anti-cancer drug targets, some patients do not respond well to tyrosine kinase inhibitors. Therefore, it is important to know which parameters lead to this lack of response. For example, demographic, genetic and clinical conditions of patients may affect the abundance/activity of RTKs, and hence, response to their inhibitors. In this study, sex, BMI and anatomical source of biopsies/samples did not have an effect on abundance. However, age correlated with several RTKs. Another important parameter that may affect the response of patients to RTK inhibitors is the abundance of DMEs and transporters involved in the metabolism and disposition of these drugs. Our previous study (30) quantified DMEs and transporters in the same sets of samples. Based on these data, correlations between the abundance of RTKs and levels of DMEs and transporters was further investigated. Our analysis highlighted several significant correlations of DMEs and transporters with RTKs. Regorafenib is a drug used in the treatment of CRLM and is metabolised by CYP3A4 and UGT1A9 (62) and transported by MRP2 and OATP1B1 (63). The implementation of these correlations should allow more realistic virtual populations to be generated, allowing better prediction of exposure and response to drugs, especially in the extreme cases of adverse reactions or lack of therapeutic efficacy (64). Further correlation between PD markers (e.g., RTKs) and PK markers (enzymes and transporters) is novel and should allow the right treatment and dose adjustments to be made for individuals. A limitation of this study is that the total number of samples and the number of samples where the RTKs were quantified are small. Suitable tissue is difficult to obtain because of the lack of a suitable, simple ethical framework for obtaining tissue samples following surgery or post-mortem. Thus, this study provided for the first time absolute quantification of RTKs in human livers. To our knowledge, there are no other studies providing such data and measurements in 15 healthy controls compared with 0 is a gain. We intend to add to these data as suitable samples become available.

In conclusion, our study provides, for the first time, absolute quantification of RTKs in liver tissue from healthy individuals and cancer patients with a focus on CRLM, reflecting a significant suppression of EGFR, INSR, VGFR3, and AXL, and upregulation of IGF1R, EPHA2 and PGFRB in tumour. It is also interesting that healthy and non-tumorous (histologically normal) livers show differential kinase profiles. Samples from CRC primary tumours may be useful to assess suitablility of treatment if the same patterns are followed in colon. Several correlations among RTKs were observed in all groups of livers showing a potential interaction and regulation of common pathways.

## Data availability statement

The original contributions presented in the study are included in the article/**Supplementary Material**. Further inquiries can be directed to the corresponding author.

## Ethics statement

The studies involving human participants were reviewed and approved by NRES Committee North West - Haydock (NRES 14/NW/1260 and 19/NW/0644). The patients/participants provided their written informed consent to participate in this study.

## Author contributions

Participated in research design: A-MV, SP, JB, AR-H. Conducted experiments: A-MV. Performed data analysis: A-MV, ZA-M, BA. Wrote or contributed to the writing of the manuscript: A-MV, ZA-M, BA, SP, JB, AR-H. All authors contributed to the article and approved the submitted version.
