# Supplementary material for: Proteomic quantification of receptor tyrosine kinases involved in the development and progression of colorectal cancer liver metastasis
Source: Front Oncol. 2023 Feb 20;13:1010563. doi: 10.3389/fonc.2023.1010563 (PMC9986493; doi:10.3389/fonc.2023.1010563)
Supplement: Supplementary file 1 [file DataSheet_1.docx]

**Proteomic Quantification of Receptor Tyrosine Kinases Involved in Cancer Development and Progression in Patients with Colorectal Cancer Liver Metastasis**

Areti-Maria Vasilogianni, Zubida M. Al-Majdoub, Brahim Achour, Sheila Annie Peters, Amin Rostami-Hodjegan, Jill Barber

*Frontiers in Oncology*

**Supplementary Data**

**Supplementary Tables**

**Table S1.** Demographic and clinical information of cancer patients provided by the MFT Biobank.

| **Sample ID** | **Age at surgery (years)** | **Race** | **Sex** | **Body mass index, BMI (kg/m^2^)** | **Smoking/ Alcohol use** | **Liver lobe** | **Diagnosis** | **Medical history** | **Treatment** |
| --- | --- | --- | --- | --- | --- | --- | --- | --- | --- |
| **389** | 52 | Caucasian | Female | 30.86 | No/ Occasionally | Left | Metastatic moderately well differentiated adenocarcinoma | Deep vein thrombosis, asthma, duodenal ulcer, thyroid problem, liver lesions | Fragmin, levothyroxine, betamethasone, ventolin, ferrous fumarate |
| **590** | 72 | Caucasian | Male | 32 | Pipe/ 22 units per week | - | Metastatic moderate to Well differentiated adenocarcinoma (dirty necrosis) | Asthma, polypectomy, tonsillectomy, Hemicolectomy Dukes B | Salbutamol, tiotropium, lansaprozole, nasonex |
| **633** | 67 | Caucasian | Male | 26.85 | Ex-smoker/ - | Right | Metastatic adenocarcinoma & fatty liver disease | Peripheral neuropathy secondary to oxaliplatin, type 2 diabetes, hypercholesterolemia, valvular heart disease, prostate cancer with bone metastasis, colonic cancer T3N0, colorectal liver metastasis | Metformin, zoladex, oxaplatin and 5FU, irinotecan and 5FU with cetuximab |
| **674** | 68 | Caucasian | Female | 26.67 | No/ - | Right | Metastatic moderately differentiated adenocarcinoma | Rectosigmoid cancer 10/10 Dukes B | - |
| **734** | 64 | Caucasian | Female | 23.84 | No/ Occasionally | Right | Moderately to focally poorly differentiated metastatic adenocarcinoma | Primary colorectal | Dalteparin, short course of radiotherapy, adjuvant OXmdG and 5FU |
| **746** | 85 | Caucasian | Male | 23.67 | Ex-smoker (40 years)/ Moderately | Right | Metastatic papillary carcinoma | Laparoscopic R hemicolectomy T2M0, Squamous cell carcinoma (scalp), hypothyroidism, hypertension, Chronic obstructive pulmonary disease | Irbesartan, levothyroxine, bisoprolol, aspirin, omeprazole, budesamide, formoterol |
| **794** | 71 | Caucasian | Female | 22.41 | No/ No | - | Metastatic adenocarcinoma with extensive intra-acinar necrosis | R hemicolectomy, pT3N2, high blood pressure, depression | Tomudex chemotherapy |
| **818** | 58 | Caucasian | Male | 21.78 | Ex-smoker (25 years)/ 18 units per week | - | Moderately differentiated metastatic adenocarcinoma | Sigmoid adenocarcinoma pT3pN2 | Loperamide, carboplatin/5FU and modified de Gramont and radiotherapy |
| **1492** | 34 | - | Female | 32.53 | Ex-smoker - stopped/ Approximately 20 units per week | Right | Metastatic moderate and poorly differentiated adenocarcinoma | Bowel resection, pilonodal abcess x2, grometts (as a child), tonsillectomy (as a child), egg collection, occasional palpitations, asthma (as a child), reflux, joint problems in knees, treated for Irritable bowel syndrome | Omeprazole, amitryptyline, microgynon, glucosamine sulphate, ibuprofen, peppermint oil |
| **1493** | 75 | - | Male | - | No/ No | Right | Metastatic moderately differentiated adenocarcinoma | Sigmoid tumour, sleep apnoea, asthma | Cod liver oil, salbutamol inhaler, seretide inhaler, movicol |
| **1498** | 63 | Caucasian | Male | - | No/ Rarely | Right | Metastatic adenocarcinoma | Previous gout, anaemia, cataract operation | Doxycycline regime completed, Nil regular |
| **1795** | 63 |  | Male | 36.32 | Ex-smoker - stopped (previously 30cpd)/ Approximately 75 units per week | Left | Metastatic well differentiated adenocarcinoma | Adenocarcinoma, hypertension, intermittent claudication of left leg | Omeprazole, irbesartan, simvastatin, clopidogrel |
| **1957** | 68 | - | Male | 32.16 | No/ - | Left | Metastatic moderately differentiated adenocarcinoma | Primary rectal cancer, pneumonia post-operative, liver cancer, late lung metastasis | Nil regular |
| **2036** | 43 | - | Female | - | -/ - | Right | Metastatic moderate to poorly differentiated adenocarcinoma | Primary colorectal | Omeprazole, paracetamol |
| **2058** | 79 | Caucasian | Female | 21.6 | -/ - | Left | Metastatic adenocarcinoma | Below the knee amputation, primary colorectal, lung metastasis | Lansoprazole, ferrous sulphate, alendronic acid, paracetamol, codeine phosphate, senna, natecal D3 |
| **2095** | 55 | Caucasian | Male | 28.1 | -/ - | Right | Metastatic moderately differentiated adenocarcinoma | Primary colorectal | Nil regular |
| **1063** | 77 | Caucasian | Male | 26.6 | Ex-smoker - stopped 20 years ago/ 15 units per week | Right | Moderate to poorly differentiated hepatocellular carcinoma | Primary hepatocellular carcinoma, prostate cancer | - |
| **1359** | 68 | Caucasian | Male | 33.4 | No/ Whiskey (frequency unknown) | Left | Poorly differentiated intrahepatic cholangiocarcinoma pT2a, pN1 | Primary liver tumour,  right elbow surgery, patient would have a cholecystectomy for gallstones but surgery was abandoned when the liver tumour was discovered, hiatus hernia, reflux | Lumigan eye drops, brinzolamide, timolol, omeprazole, bimatoprost |

**Table S2.** Demographic and clinical information of healthy subjects provided by Pfizer.

| **Sample ID** | **Age at surgery (years)** | **Race** | **Sex** | **Body mass index, BMI (kg/m^2^)** | **Smoking/ Alcohol use** | **Cause of death** | **Medical history** | **Treatment** |
| --- | --- | --- | --- | --- | --- | --- | --- | --- |
| **HH83** | 18 | Caucasian | Female | 20.19 | No/No | Head trauma | Healthy | None |
| **HH84** | 53 | Caucasian | Male | 19.94 | No/Social | Intracranial haemorrhage | Healthy | None |
| **HH87** | 54 | Caucasian | Female | 29.79 | No/No | Head trauma | Healthy | None |
| **HH93** | 34 | Caucasian | Male | 20.62 | No/No | Cerebellar haemorrhagic injury | Healthy | Healthy |
| **HH98** | 64 | Caucasian | Male | 37.47 | No/No | Head Injury | Healthy | None |
| **HH99** | 45 | Caucasian | Male | 31.62 | No/No | Head trauma | Healthy | None |
| **HH101** | 54 | Caucasian | Female | 21.95 | No/No | Motor vehicle accident | Healthy | None |
| **HH102** | 52 | Caucasian | Female | 32.26 | No/No | Cerebral Aneurysm | Healthy | None |
| **HH104** | 35 | African American | Female | 25.25 | No/No | Cerebral Aneurysm | Healthy | None |
| **HH105** | 50 | Caucasian | Male | 33.47 | No/No | Cerebral Aneurysm | Healthy | None |
| **HH106** | 43 | Hispanic | Male | 24.48 | No/No | Cerebral Vascular Aneurysm | Healthy | None |
| **HH107** | 45 | Caucasian | Female | 24.96 | No/No | Cerebral Vascular Aneurysm | Healthy | None |
| **HH110** | 54 | Caucasian | Female | 26.29 | No/Social | Cerebral Vascular Aneurysm | Healthy | None |
| **HH111** | 43 | Caucasian | Female | 28.43 | No/No | Intracranial bleeding | Healthy | None |
| **HH118** | 32 | Caucasian | Male | 26.69 | No/Social | Gunshot Wound to head | Healthy, Skin graft on right arm in the past | Pepcid AC, Tagamet, Steroids in HS and Marines |

**Table S3.** Abundance levels of 21 RTKs in the healthy, non-tumorous (histologically normal) and tumorous sample sets represented by the median, mean, standard deviation of the mean (SD), coefficient of variation (CV), and range (minimum to maximum).

| **RTK** | **Median (pmol mg^−1^)** | | | **Mean ± SD (pmol mg^−1^)** | | | **CV (%)** | | | **Range (pmol mg^−1^)** | | | **n** | | |
| --- | --- | --- | --- | --- | --- | --- | --- | --- | --- | --- | --- | --- | --- | --- | --- |
|  | **H** | **N** | **T** | **H** | **N** | **T** | **H** | **N** | **T** | **H** | **N** | **T** | **H** | **N** | **T** |
| **AXL**  (tyrosine-protein kinase receptor UFO) | 0.05 | 0.04 | 0.02 | 0.06 ± 0.04 | 0.04 ± 0.02 | 0.03 ± 0.03 | 64 | 66 | 79 | 0.02 - 0.15 | 0.01 - 0.08 | 0.003 - 0.1 | 14 | 15 | 15 |
| **CSF1R** (macrophage colony-stimulating factor 1 receptor) | 0.22 | 0.10 | 0.10 | 0.22 ± 0.11 | 0.1 ± 0.09 | 0.18 ± 0.19 | 49 | 95 | 111 | 0.11 - 0.38 | 0.03 - 0.16 | 0.04 - 0.7 | 5 | 2 | 10 |
| **EGFR** (epidermal growth factor receptor) | 0.31 | 0.19 | 0.08 | 0.34 ± 0.15 | 0.22 ± 0.12 | 0.09 ± 0.06 | 43 | 56 | 62 | 0.15 - 0.57 | 0.02 - 0.61 | 0.01 - 0.19 | 15 | 18 | 18 |
| **EPHA2** (ephrin type-A receptor 2) | 0.04 | 0.04 | 0.06 | 0.05 ± 0.02 | 0.04 ± 0.03 | 0.09 ± 0.09 | 45 | 69 | 90 | 0.04 - 0.08 | 0.002 - 0.08 | 0.02 - 0.34 | 3 | 9 | 14 |
| **ERBB2** (human epidermal growth factor receptor 2) | 0.05 | 0.04 | 0.05 | 0.05 ± 0.03 | 0.05 ± 0.03 | 0.06 ± 0.04 | 61 | 69 | 68 | 0.02 - 0.13 | 0.001 - 0.14 | 0.02 - 0.14 | 9 | 15 | 10 |
| **FGFR1** (fibroblast growth factor receptor 1) | 0.36 | 0.17 | 0.13 | 0.58 ± 0.59 | 0.36 ± 0.51 | 0.13 ± 0.03 | 103 | 144 | 20 | 0.15 - 1.45 | 0.05 - 1.69 | 0.11 - 0.15 | 4 | 9 | 2 |
| **FGFR2** (fibroblast growth factor receptor 2) | 0.12 | 0.07 | 0.07 | 0.12 ± 0.07 | 0.05 ± 0.03 | 0.09 ± 0.08 | 58 | 64 | 83 | 0.01 - 0.25 | 0.01 - 0.12 | 0.001 - 0.25 | 14 | 13 | 15 |
| **FGFR3** (fibroblast growth factor receptor 3) | 0.03 | 0.02 | 0.03 | 0.03 ± 0.01 | 0.03 ± 0.03 | 0.04 ± 0.03 | 49 | 94 | 82 | 0.01 - 0.06 | 0.002 - 0.1 | 0.0003 - 0.11 | 8 | 13 | 11 |
| **FLT3** (FMS-like tyrosine kinase) | - | - | 0.05 | - | - | - | - | - | - | - | - | - | - | - | 1 |
| **IGF1R** (insulin-like growth factor 1 receptor) | 0.06 | 0.02 | 0.08 | 0.09 ± 0.07 | 0.04 ± 0.03 | 0.08 ± 0.04 | 84 | 95 | 45 | 0.03 - 0.23 | 0.01 - 0.11 | 0.04 - 0.13 | 7 | 8 | 6 |
| **INSR** (insulin receptor) | 0.37 | 0.12 | 0.12 | 0.39 ± 0.11 | 0.14 ± 0.09 | 0.13 ± 0.06 | 29 | 67 | 45 | 0.17 - 0.59 | 0.03 - 0.35 | 0.01 - 0.24 | 15 | 17 | 18 |
| **KIT** (mast/stem cell growth factor receptor) | 0.18 | 0.16 | 0.44 | 0.19 ± 0.11 | 0.23 ± 0.23 | - | 56 | 101 | - | 0.03 - 0.41 | 0.003 - 0.77 | - | 9 | 10 | 1 |
| **MET** (hepatocyte growth factor receptor) | - | - | 0.39 | - | - | 0.39 ± 0.14 | - | - | 36 | - | - | 0.29 - 0.49 | - | - | 2 |
| **NTRK2** (neurotrophic tyrosine kinase receptor type 2) | 0.06 | 0.04 | 0.04 | 0.1 ± 0.15 | 0.06 ± 0.06 | 0.05 ± 0.04 | 145 | 103 | 72 | 0 - 0.54 | 0.01 - 0.28 | 0.01 - 0.13 | 11 | 16 | 14 |
| **PGFRA** (platelet-derived growth factor receptor A) | 0.07 | 0.04 | 0.03 | 0.07 ± 0.04 | 0.04 ± 0.04 | 0.05 ± 0.04 | 60 | 81 | 86 | 0.01 - 0.15 | 0.0004 - 0.14 | 0.01 - 0.13 | 15 | 16 | 12 |
| **PGFRB** (platelet-derived growth factor receptor B) | 0.11 | 0.12 | 2.18 | 0.11 ± 0.04 | 0.13 ± 0.06 | 2.2 ± 1.85 | 34 | 43 | 84 | 0.07 - 0.17 | 0.09 - 0.21 | 0.6 - 5.91 | 8 | 4 | 7 |
| **RET** (proto-oncogene tyrosine-protein kinase receptor) | 1.19 | 0.98 | 0.30 | 1.48 ± 0.92 | 0.98 ± 0.61 | 0.3 ± 0.32 | 62 | 62 | 104 | 0.55 - 2.79 | 0.03 - 1.73 | 0.08 - 0.53 | 5 | 7 | 2 |
| **TIE2** (angiopoietin-1 receptor) | 0.16 | 0.11 | 0.18 | 0.13 ± 0.06 | 0.12 ± 0.06 | 0.24 ± 0.19 | 42 | 44 | 79 | 0.06 - 0.21 | 0.05 - 0.22 | 0.08 - 0.52 | 9 | 14 | 4 |
| **VGFR1** (vascular endothelial growth factor receptor 1) | 0.06 | 0.01 | 0.09 | 0.07 ± 0.05 | 0.11 ± 0.17 | - | 80 | 158 | - | 0.01 - 0.14 | 0.01 - 0.3 | - | 8 | 3 | 1 |
| **VGFR2** (vascular endothelial growth factor receptor 2) | 0.09 | 0.03 | 0.02 | 0.14 ± 0.16 | 0.02 ± 0.02 | 0.02 ± 0.01 | 119 | 72 | 73 | 0.01 - 0.32 | 0.001 - 0.04 | 0.003 - 0.03 | 3 | 6 | 4 |
| **VGFR3** (vascular endothelial growth factor receptor 3) | 0.06 | 0.03 | 0.03 | 0.07 ± 0.03 | 0.05 ± 0.06 | 0.05 ± 0.08 | 49 | 111 | 177 | 0.04 - 0.13 | 0.01 - 0.21 | 0.004 - 0.28 | 9 | 13 | 11 |

**Table S4.** Targeted quantification of 19 receptor tyrosine kinases (RTKs) in 15 human liver microsomes from healthy subjects. In red font are measurements that are below the lower limit of quantification. In cases where values are missing, the peptides were not identified and quantified.

| **Sample ID by the provider** | | **HH83** | **HH84** | **HH87** | **HH93** | **HH98** | **HH99** | **HH101** | **HH102** | **HH104** | **HH105** | **HH106** | **HH107** | **HH110** | **HH111** | **HH118** |
| --- | --- | --- | --- | --- | --- | --- | --- | --- | --- | --- | --- | --- | --- | --- | --- | --- |
| **Sample ID** | | **H3** | **H6** | **H9** | **H12** | **H51** | **H18** | **H21** | **H53** | **H27** | **H30** | **H33** | **H36** | **H39** | **H42** | **H56** |
| **Protein target** | **Peptides** | **Absolute abundance (pmol mg^-1^)** | | | | | | | | | | | | | | |
| VGFR2 | AASVGLPSVSLDLPR | 0.317 | 0.005 |  |  | 0.087 |  |  |  |  |  |  |  |  |  |  |
| RET | LLEGEGLPFR | 2.023 | 1.185 | 2.793 |  |  |  | 0.545 |  |  |  | 0.854 |  |  |  |  |
| FGFR1 | DGVQLAESNR |  |  |  |  | 0.284 |  |  | 0.426 | 0.145 |  |  |  |  |  | 1.448 |
| EGFR | IPLENLQIIR | 0.211 | 0.197 | 0.307 | 0.314 | 0.204 | 0.573 | 0.569 | 0.306 | 0.147 | 0.502 | 0.185 | 0.356 | 0.549 | 0.314 | 0.356 |
| INSR | ESLVISGLR | 0.292 | 0.367 | 0.372 | 0.472 | 0.373 | 0.435 | 0.366 | 0.348 | 0.165 | 0.474 | 0.242 | 0.297 | 0.589 | 0.474 | 0.539 |
| NTRK2 | NSNLQHINFTR | 0.038 | 0.042 |  |  | 0.002 |  |  | 0.032 | 0.081 | 0.063 | 0.197 | 0.536 | 0.058 | 0.027 | 0.078 |
| AXL | APLQGTLLGYR | 0.076 | 0.073 | 0.042 | 0.047 | 0.043 | 0.019 | 0.133 |  | 0.037 | 0.017 | 0.023 | 0.087 | 0.069 | 0.145 | 0.052 |
| VGFR3 | NILLSESDVVK | 0.037 | 0.071 |  | 0.102 | 0.038 | 0.046 |  | 0.132 | 0.044 |  |  |  | 0.060 | 0.057 |  |
| PGFRA | VVEGTAYGLSR | 0.078 | 0.076 | 0.092 | 0.014 | 0.055 | 0.154 | 0.050 | 0.077 | 0.043 | 0.076 | 0.016 | 0.034 | 0.136 | 0.029 | 0.073 |
| FGFR2 | EIEVLYIR | 0.119 | 0.114 | 0.168 | 0.086 | 0.181 | 0.224 | 0.146 | 0.136 | 0.027 | 0.011 | 0.063 | 0.115 | 0.248 | 0.051 |  |
| CSF1R | VVEATAFGLGK |  | 0.133 |  | 0.377 |  |  | 0.111 |  | 0.220 | 0.282 |  |  |  |  |  |
| IGF1R | TTINNEYNYR |  |  |  | 0.228 | 0.137 |  |  | 0.034 | 0.027 | 0.033 |  | 0.062 | 0.090 |  |  |
| ERBB2 | LLDIDETEYHADGGK | 0.024 | 0.057 | 0.046 |  | 0.081 |  | 0.128 | 0.054 | 0.049 |  |  | 0.033 | 0.018 |  |  |
| FGFR3 | VGPDGTPYVTVLK |  | 0.025 |  | 0.022 | 0.031 | 0.014 | 0.042 | 0.030 |  | 0.017 |  |  | 0.059 |  |  |
| VGFR1 | FNSGSSDDVR |  |  |  | 0.120 | 0.112 |  | 0.021 | 0.142 | 0.016 | 0.083 | 0.005 |  | 0.038 |  |  |
| EPHA2 | TVSEWLESIK |  |  |  |  |  |  | 0.042 |  |  | 0.039 |  |  | 0.083 |  |  |
| TIE2 | NILVGENYVAK |  | 0.168 | 0.159 | 0.058 | 0.206 | 0.193 |  |  | 0.064 | 0.089 | 0.095 | 0.155 |  |  |  |
| KIT | LVVQSSIDSSAFK |  | 0.034 | 0.177 | 0.143 | 0.133 |  | 0.413 | 0.249 | 0.128 |  | 0.233 |  | 0.200 |  |  |
| PGFRB | GFSGIFEDR |  |  |  | 0.169 | 0.073 | 0.067 | 0.145 |  | 0.155 |  | 0.084 | 0.114 | 0.100 |  |  |
| FLT3 | TWTEIFK |  |  |  |  |  |  |  |  |  |  |  |  |  |  |  |
| MET | LNSELNIEWK |  |  |  |  |  |  |  |  |  |  |  |  |  |  |  |

**Table S5.** Targeted quantification of 19 receptor tyrosine kinases (RTKs) in 18 non-tumorous (histologically normal) liver microsomes from cancer patients. In red font are measurements that are below the lower limit of quantification. In cases where values are missing, the peptides were not identified and quantified.

| **Sample ID by the provider** | | **2095** | **2058** | **2036** | **389** | **590** | **746** | **818** | **1492** | **674** | **1957** | **1493** | **1498** | **633** | **734** | **794** | **1795** | **1063** | **1359** |
| --- | --- | --- | --- | --- | --- | --- | --- | --- | --- | --- | --- | --- | --- | --- | --- | --- | --- | --- | --- |
| **Sample ID** | | **N1** | **N4** | **N7** | **N10** | **N13** | **N49** | **N19** | **N22** | **N25** | **N34** | **N28** | **N31** | **N37** | **N40** | **N43** | **N54** | **N45** | **N47** |
| **Protein target** | **Peptides** | **Absolute abundance (pmol mg^-1^)** | | | | | | | | | | | | | | | | | |
| VGFR2 | AASVGLPSVSLDLPR |  |  | 0.022 | 0.042 |  |  |  | 0.006 | 0.035 |  | 0.030 |  |  |  | 0.001 |  |  |  |
| RET | LLEGEGLPFR |  | 1.730 |  | 0.723 |  |  |  | 0.029 | 1.673 |  | 1.131 |  | 0.565 |  |  |  | 0.979 |  |
| FGFR1 | DGVQLAESNR |  |  |  |  |  |  | 0.354 | 0.168 |  |  | 0.393 |  | 0.149 | 0.193 | 0.047 | 1.686 | 0.064 | 0.143 |
| EGFR | IPLENLQIIR | 0.283 | 0.145 | 0.173 | 0.185 | 0.249 | 0.224 | 0.613 | 0.265 | 0.307 | 0.170 | 0.138 | 0.198 | 0.166 | 0.345 | 0.093 | 0.245 | 0.024 | 0.171 |
| INSR | ESLVISGLR | 0.132 | 0.075 | 0.076 | 0.135 | 0.257 | 0.088 | 0.352 | 0.230 | 0.104 | 0.063 | 0.126 | 0.188 | 0.057 | 0.310 | 0.066 |  | 0.119 | 0.028 |
| NTRK2 | NSNLQHINFTR | 0.098 | 0.011 | 0.018 | 0.023 |  | 0.276 |  | 0.021 | 0.061 | 0.023 | 0.079 | 0.018 | 0.043 | 0.087 | 0.072 | 0.099 | 0.035 | 0.037 |
| AXL | APLQGTLLGYR |  | 0.052 | 0.036 | 0.025 | 0.082 | 0.023 | 0.068 | 0.030 | 0.054 |  | 0.036 | 0.016 | 0.037 | 0.056 | 0.005 | 0.005 |  | 0.006 |
| VGFR3 | NILLSESDVVK | 0.075 | 0.010 | 0.061 | 0.021 | 0.209 |  |  | 0.009 |  | 0.018 | 0.127 | 0.044 |  | 0.053 | 0.026 |  | 0.006 | 0.020 |
| PGFRA | VVEGTAYGLSR | 0.081 | 0.037 | 0.009 | 0.042 | 0.043 | 0.143 | 0.049 | 0.061 |  | 0.021 | 0.028 | 0.018 | 0.020 | 0.085 | 0.035 | 0.024 |  | 0.000 |
| FGFR2 | EIEVLYIR |  | 0.066 | 0.085 | 0.065 | 0.115 | 0.072 |  | 0.074 | 0.073 | 0.022 | 0.033 | 0.014 |  | 0.008 | 0.026 |  |  | 0.021 |
| CSF1R | VVEATAFGLGK |  |  |  |  |  |  | 0.164 |  |  |  |  |  |  |  | 0.032 |  |  |  |
| IGF1R | TTINNEYNYR |  | 0.017 |  |  | 0.108 |  | 0.043 | 0.011 | 0.014 |  | 0.010 |  | 0.026 |  | 0.052 |  |  |  |
| ERBB2 | LLDIDETEYHADGGK | 0.041 |  | 0.066 | 0.061 |  |  | 0.137 | 0.057 | 0.050 | 0.034 | 0.017 | 0.014 | 0.039 | 0.040 | 0.001 | 0.097 | 0.038 | 0.039 |
| FGFR3 | VGPDGTPYVTVLK | 0.102 | 0.032 |  | 0.020 |  |  |  | 0.028 | 0.008 | 0.023 | 0.011 | 0.030 | 0.014 | 0.038 | 0.028 |  | 0.002 | 0.010 |
| VGFR1 | FNSGSSDDVR |  |  |  |  |  | 0.298 |  |  |  |  |  |  | 0.012 |  | 0.006 |  |  |  |
| EPHA2 | TVSEWLESIK |  |  |  | 0.016 |  |  | 0.002 |  | 0.021 |  | 0.081 | 0.041 | 0.040 | 0.068 | 0.021 | 0.042 |  |  |
| TIE2 | NILVGENYVAK | 0.205 | 0.060 | 0.224 | 0.121 |  | 0.054 | 0.156 | 0.156 | 0.084 |  | 0.196 | 0.090 |  | 0.118 | 0.068 |  | 0.101 | 0.108 |
| KIT | LVVQSSIDSSAFK |  |  |  | 0.152 | 0.401 | 0.309 | 0.773 | 0.169 |  |  | 0.160 |  | 0.219 |  | 0.003 |  | 0.064 | 0.015 |
| PGFRB | GFSGIFEDR |  |  |  | 0.208 |  |  | 0.092 | 0.144 |  |  | 0.086 |  |  |  |  |  |  |  |
| FLT3 | TWTEIFK |  |  |  |  |  |  |  |  |  |  |  |  |  |  |  |  |  |  |
| MET | LNSELNIEWK |  |  |  |  |  |  |  |  |  |  |  |  |  |  |  |  |  |  |

**Table S6.** Targeted quantification of 21 receptor tyrosine kinases (RTKs) in 18 tumorous human liver microsomes from cancer patients. In red font are measurements that are below the lower limit of quantification. In cases where values are missing, the peptides were not identified and quantified.

| **Sample ID by the provider** | | **2095** | **2058** | **2036** | **389** | **590** | **746** | **818** | **1492** | **674** | **1957** | **1493** | **1498** | **633** | **734** | **794** | **1795** | **1063** | **1359** |
| --- | --- | --- | --- | --- | --- | --- | --- | --- | --- | --- | --- | --- | --- | --- | --- | --- | --- | --- | --- |
| **Sample ID** | | **T2** | **T5** | **T8** | **T11** | **T14** | **T50** | **T52** | **T23** | **T26** | **T35** | **T29** | **T32** | **T38** | **T41** | **T44** | **T55** | **T46** | **T48** |
| **Protein target** | **Peptides** | **Absolute abundance (pmol mg^-1^)** | | | | | | | | | | | | | | | | | |
| VGFR2 | AASVGLPSVSLDLPR |  |  |  |  |  | 0.020 |  |  | 0.010 |  |  | 0.029 |  |  |  |  |  | 0.003 |
| RET | LLEGEGLPFR |  |  |  | 0.080 |  |  |  |  |  |  |  | 0.529 |  |  |  |  |  |  |
| FGFR1 | DGVQLAESNR |  |  |  |  |  |  |  |  |  | 0.151 |  |  |  | 0.113 |  |  |  |  |
| EGFR | IPLENLQIIR | 0.008 | 0.067 | 0.084 | 0.080 | 0.108 | 0.175 | 0.044 | 0.136 | 0.149 | 0.120 | 0.018 | 0.164 | 0.030 | 0.054 | 0.070 | 0.097 | 0.033 | 0.189 |
| INSR | ESLVISGLR | 0.171 | 0.114 | 0.135 | 0.095 | 0.236 | 0.177 | 0.110 | 0.103 | 0.184 | 0.200 | 0.121 | 0.086 | 0.211 | 0.112 | 0.059 | 0.218 | 0.014 | 0.079 |
| NTRK2 | NSNLQHINFTR |  |  |  | 0.063 | 0.035 | 0.093 | 0.033 | 0.012 | 0.034 | 0.006 | 0.065 | 0.126 | 0.042 | 0.053 | 0.014 | 0.096 |  | 0.019 |
| AXL | APLQGTLLGYR | 0.024 | 0.011 | 0.035 | 0.046 | 0.022 | 0.102 | 0.023 | 0.016 |  | 0.003 |  | 0.034 | 0.034 | 0.017 |  | 0.076 | 0.011 | 0.066 |
| VGFR3 | NILLSESDVVK | 0.284 | 0.030 |  | 0.028 | 0.017 |  | 0.036 |  | 0.016 | 0.004 | 0.008 |  |  | 0.025 | 0.023 | 0.026 |  |  |
| PGFRA | VVEGTAYGLSR | 0.024 |  |  | 0.011 | 0.131 | 0.128 | 0.053 | 0.021 |  |  | 0.024 | 0.028 | 0.038 | 0.039 | 0.086 |  | 0.011 |  |
| FGFR2 | EIEVLYIR | 0.085 | 0.112 | 0.249 | 0.104 | 0.208 | 0.198 |  | 0.072 | 0.048 | 0.001 | 0.027 | 0.074 | 0.130 | 0.007 | 0.047 |  |  | 0.019 |
| CSF1R | VVEATAFGLGK |  | 0.080 | 0.697 | 0.260 | 0.087 |  | 0.102 |  | 0.044 |  | 0.103 | 0.174 | 0.141 | 0.063 |  |  |  |  |
| IGF1R | TTINNEYNYR |  |  |  | 0.042 | 0.118 |  | 0.062 |  |  | 0.045 |  | 0.092 | 0.126 |  |  |  |  |  |
| ERBB2 | LLDIDETEYHADGGK | 0.040 | 0.128 | 0.028 | 0.079 | 0.139 | 0.045 | 0.054 | 0.031 |  |  |  | 0.022 |  | 0.046 |  |  |  |  |
| FGFR3 | VGPDGTPYVTVLK | 0.107 |  | 0.063 | 0.0003 | 0.021 | 0.020 | 0.018 | 0.066 |  | 0.051 | 0.001 | 0.032 | 0.069 |  |  |  |  |  |
| VGFR1 | FNSGSSDDVR |  |  |  |  |  |  |  | 0.094 |  |  |  |  |  |  |  |  |  |  |
| EPHA2 | TVSEWLESIK |  | 0.056 |  | 0.023 | 0.117 | 0.161 | 0.039 | 0.049 | 0.344 | 0.036 | 0.055 |  | 0.071 | 0.119 | 0.027 | 0.165 |  | 0.066 |
| TIE2 | NILVGENYVAK |  | 0.515 |  | 0.084 |  |  | 0.205 |  |  |  |  | 0.160 |  |  |  |  |  |  |
| KIT | LVVQSSIDSSAFK |  |  |  |  |  |  |  |  |  |  |  | 0.438 |  |  |  |  |  |  |
| PGFRB | GFSGIFEDR |  |  |  |  |  |  | 0.904 | 0.598 | 2.516 |  | 5.914 |  | 2.181 |  |  | 2.582 |  | 0.693 |
| FLT3 | TWTEIFK |  |  |  |  |  |  |  |  |  | 0.054 |  |  |  |  |  |  |  |  |
| MET | LNSELNIEWK |  |  |  |  | 0.487 | 0.290 |  |  |  |  |  |  |  |  |  |  |  |  |

**Table S7.** Correlations (Spearman coefficient, Rs) of protein abundance between receptor tyrosine kinases (RTKs) and cytochrome P450 (CYP) enzymes, UDP-glucuronosyltransferases (UGT), ATP-binding cassette (ABC) transporters and solute carriers (SLC).

|  | **VGFR2** | **RET** | **FGFR1** | **EGFR** | **INSR** | **NTRK2** | **AXL** | **VGFR3** | **PGFRA** | **FGFR2** | **CSF1R** | **IGF1R** | **ERBB2** | **FGFR3** | **VGFR1** | **EPHA2** | **TIE2** | **KIT** | **PGFRB** |
| --- | --- | --- | --- | --- | --- | --- | --- | --- | --- | --- | --- | --- | --- | --- | --- | --- | --- | --- | --- |
| **CYP1A2** | - | Rs = 0.61, *p* = 0.03 | - | Rs = 0.58, *p* < 0.001 | - | - | Rs = 0.45, *p* = 0.003 | - | - | - | - | - | - | - | - | - | - | - | Rs = -0.51, *p* = 0.05 |
| **CYP2A6** | Rs = 0.84, *p* = 0.001 | - | - | Rs = 0.53, *p* < 0.001 | - | - | - | - | - | - | - | - | - | - | - | - | Rs = -0.42, *p* = 0.03 | - | Rs = -0.56, *p* = 0.03 |
| **CYP2B6** | Rs = 0.93, *p* = 0.002 | - | - | Rs = 0.59, *p* = 0.003 | Rs = 0.55, *p* = 0.007 | - | - | - | - | - | - | - | - | - | - | - | - | Rs = 0.56, *p* = 0.04 | - |
| **CYP2C18** | - | - | - | Rs = 0.38, *p* = 0.04 | - | - | - | Rs = 0.55, *p* = 0.02 | - | - | - | - | - | - | - | Rs = -0.54, *p* = 0.05 | - | - | - |
| **CYP2C19** | - | - | - | Rs = 0.52, *p* < 0.001 | - | - | Rs = 0.41, *p* = 0.009 | - | - | - | - | - | - | - | - | Rs = -0.51, *p* = 0.02 | - | - | - |
| **CYP2C8** | Rs = 0.81, *p* = 0.002 | - | - | Rs = 0.48, *p* = 0.002 | Rs = 0.46, *p* = 0.003 | - | - | - | Rs = 0.36, *p* = 0.03 | - | - | - | - | - | - | - | - | Rs = 0.45, *p* = 0.05 | Rs = -0.66, *p* = 0.009 |
| **CYP2C9** | - | - | - | Rs = 0.69, *p* < 0.001 | Rs = 0.47, *p* = 0.001 | - | Rs = 0.32, *p* = 0.04 | Rs = 0.52, *p* = 0.003 | - | - | - | - | - | - | - | - | - | - | Rs = -0.89, *p* < 0.001 |
| **CYP2D6** | - | - | - | Rs = 0.83, *p* < 0.001 | Rs = 0.66, *p* < 0.001 | - | - | Rs = 0.56, *p* = 0.01 | - | - | - | - | - | - | - | - | - | - | Rs = -0.77, *p* = 0.02 |
| **CYP2E1** | - | - | Rs = 0.5643, *p* = 0.03 | Rs = 0.77, *p* < 0.001 | Rs = 0.58, *p* < 0.001 | - | Rs = 0.35, *p* = 0.02 | Rs = 0.50, *p* = 0.003 | Rs = 0.33, *p* = 0.03 | - | Rs = 0.58, *p* = 0.02 | - | - | - | - | - | - | - | Rs = -0.77, *p* < 0.001 |
| **CYP2J2** | - | - | - | Rs = 0.68, *p* < 0.001 | Rs = 0.55, *p* < 0.001 | - | Rs = 0.37, *p* = 0.03 | Rs = 0.46, *p* = 0.02 | - | - | - | - | - | - | - | - | - | - | Rs = -0.68, *p* = 0.007 |
| **CYP3A4** | Rs = 0.62, *p* = 0.03 | - | - | Rs = 0.66, *p* < 0.001 | Rs = 0.44, *p* = 0.002 | - | Rs = 0.41, *p* = 0.005 | - | - | - | - | - | - | - | - | - | - | - | Rs = -0.72, *p* < 0.001 |
| **CYP3A5** | - | Rs = 0.71, *p* = 0.01 | - | - | - | - | - | - | - | - | - | - | - | - | - | - | - | - | - |
| **CYP3A7** | - | - | - | Rs = 0.48, *p* = 0.04 | - | - | - | Rs = 0.72, *p* = 0.008 | - | - | - | - | - | - | - | - | - | Rs = 0.81, *p* = 0.003 | - |
| **CYP4F2** | - | - | Rs = 0.55, *p* = 0.04 | Rs = 0.67, *p* < 0.001 | Rs = 0.43, *p* = 0.002 | - | Rs = 0.34, *p* = 0.03 | Rs = 0.42, *p* = 0.01 | - | - | - | - | - | - | - | Rs = -0.49, *p* = 0.01 | - | - | Rs = -0.7, *p* = 0.002 |
| **UGT1A1** | - | - | - | Rs = 0.61, *p* < 0.001 | Rs = 0.51, *p* = 0.004 | - | Rs = 0.43, *p* = 0.02 | Rs = 0.53, *p* = 0.007 | - | - | Rs = 0.67, *p* = 0.03 | - | Rs = -0.56, *p* = 0.009 | - | - | - | - | - | - |
| **UGT1A3** | - | - | - | Rs = 0.51, *p* < 0.001 | Rs = 0.34, *p* = 0.03 | - | - | - | - | - | Rs = 0.59, *p* = 0.03 | - | - | - | - | - | - | - | Rs = -0.72, *p* = 0.002 |
| **UGT1A6** | - | - | Rs = 0.67, *p* = 0.01 | Rs = 0.71, *p* < 0.001 | Rs = 0.63, *p* < 0.001 | - | - | Rs = 0.64, *p* < 0.001 | - | - | Rs = 0.71, *p* = 0.005 | - | - | - | - | - | - | - | Rs = -0.82, *p* < 0.001 |
| **UGT1A9** | - | - | Rs = 0.57, *p* = 0.03 | Rs = 0.74, *p* < 0.001 | Rs = 0.57, *p* < 0.001 | - | Rs = 0.46, *p* = 0.003 | Rs = 0.43, *p* = 0.02 | - | - | Rs = 0.63, *p* = 0.01 | - | - | - | - | - | - | - | Rs = -0.67, *p* = 0.006 |
| **UGT2B11** | - | - | - | - | - | - | - | - | - | - | - | - | - | - | - | - | - | - | - |
| **UGT2B15** | - | - | Rs = 0.60, *p* = 0.02 | Rs = 0.73, *p* < 0.001 | Rs = 0.61, *p* < 0.001 | - | Rs = 0.4, *p* = 0.01 | Rs = 0.53, *p* = 0.004 | - | - | Rs = 0.7, *p* = 0.02 | - | - | - | - | - | - | - | Rs = -0.66, *p* = 0.02 |
| **UGT2B4** | - | - | Rs = 0.79, *p* = 0.04 | Rs = 0.78, *p* < 0.001 | Rs = 0.65, *p* = 0.003 | - | - | Rs = 0.62, *p* = 0.01 | - | - | - | - | - | - | - | - | - | Rs = 0.86, *p* = 0.02 | - |
| **UGT2B7** | Rs = 0.73, *p* = 0.006 | - | Rs = 0.56, *p* = 0.03 | Rs = 0.74, *p* < 0.001 | Rs = 0.47, *p* < 0.001 | - | - | Rs = 0.45, *p* = 0.009 | - | - | Rs = 0.52, *p* = 0.04 | - | - | - | - | Rs = -0.49, *p* = 0.01 | - | - | Rs = -0.77, *p* < 0.001 |
| **BCRP** | - | - | - | - | - | - | - | - | - | - | - | - | - | - | - | - | - | - | - |
| **P-gp** | - | - | Rs = 0.7, *p* = 0.02 | Rs = 0.43, *p* = 0.01 | Rs = 0.55, *p* < 0.001 | - | - | Rs = 0.43, *p* = 0.04 | Rs = 0.36, *p* = 0.04 | Rs = 0.43, *p* = 0.02 | - | - | - | - | - | Rs = 0.61, *p* = 0.01 | - | - | - |
| **BSEP** | - | - | Rs = 0.71, *p* = 0.004 | Rs = 0.77, *p* < 0.001 | Rs = 0.61, *p* < 0.001 | - | Rs = 0.44, *p* = 0.005 | Rs = 0.55, *p* = 0.002 | - | - | - | - | - | - | - | - | - | Rs = 0.5, *p* = 0.02 | - |
| **MDR3** | - | - | - | - | - | - | - | - | - | - | - | - | - | - | - | - | - | - | - |
| **MRP2** | - | - | Rs = 0.68, *p* = 0.006 | Rs = 0.64, *p* < 0.001 | Rs = 0.53, *p* < 0.001 | - | Rs = 0.31, *p* = 0.05 | Rs = 0.5, *p* = 0.005 | - | Rs = 0.34, *p* = 0.04 | - | - | - | - | - | - | - | - | Rs = -0.51, *p* = 0.05 |
| **MRP3** | - | - | Rs = 0.79, *p* < 0.001 | Rs = 0.41, *p* = 0.002 | Rs = 0.68, *p* < 0.001 | - | - | Rs = 0.4, *p* = 0.02 | Rs = 0.36, *p* = 0.02 | Rs = 0.32, *p* = 0.04 | - | - | - | - | - | - | - | - | - |
| **MRP4** | - | - | - | Rs = 0.59, *p* = 0.02 | - | Rs = 0.68, *p* = 0.04 | - | Rs = 0.79, *p* = 0.006 | - | Rs = 0.6, *p* = 0.04 | - | - | - | - | - | - | - | - | - |
| **MRP6** | - | - | Rs = 0.76, *p* = 0.006 | Rs = 0.68, *p* < 0.001 | Rs = 0.63, *p* < 0.001 | Rs = 0.45, *p* = 0.02 | Rs = 0.65, *p* < 0.001 | Rs = 0.71, *p* < 0.001 | Rs = 0.45, *p* = 0.01 | Rs = 0.66, *p* < 0.001 | - | - | - | - | - | - | - | Rs = 0.57, *p* = 0.02 | - |
| **MATE1** | - | - | - | - | - | - | - | - | - | - | - | - | - | - | - | - | - | - | - |
| **MCT1** | - | - | - | - | Rs = 0.5, *p* = 0.003 | - | - | - | - | - | - | - | - | Rs = 0.55, *p* = 0.008 | Rs = 0.79, p = 0.05 | Rs = 0.68, *p* = 0.004 | - | - | - |
| **OSTA** | - | - | - | - | - | - | - | - | - | - | - | - | - | - | - | - | - | - | - |
| **OSTB** | - | - | - | - | - | - | Rs = -0.47, *p* = 0.03 | - | - | - | - | - | - | - | - | - | - | - | - |
| **ASBT** | - | - | - | - | - | - | - | - | - | - | - | - | - | - | - | - | - | - | - |
| **PEPT1** | - | - | - | - | - | - | - | - | - | - | - | - | - | - | - | - | - | - | - |
| **NTCP** | - | - | Rs = 0.83, *p* = 0.02 | Rs = 0.77, *p* < 0.001 | Rs = 0.74, *p* < 0.001 | - | - | - | - | - | - | - | Rs = 0.5, *p* = 0.04 | - | - | - | - | Rs = 0.81, *p* = 0.02 | - |
| **OCT1** | - | Rs = 0.7833, *p* = 0.02 | - | Rs = 0.76, *p* < 0.001 | Rs = 0.52, *p* = 0.03 | - | - | - | - | - | - | - | - | - | - | - | - | - | - |
| **OCT3** | Rs = 0.74, *p* = 0.05 | - | Rs = 0.86, *p* = 0.02 | Rs = 0.66, *p* < 0.001 | Rs = 0.63, *p* < 0.001 | - | - | - | Rs = 0.5, *p* = 0.01 | - | - | - | - | - | - | - | - | - | Rs = -0.85, *p* = 0.003 |
| **OAT2** | Rs = 0.62, *p* = 0.03 | - | Rs = 0.62, *p* = 0.02 | Rs = 0.72, *p* < 0.001 | Rs = 0.5, *p* < 0.001 | - | Rs = 0.38, *p* = 0.02 | Rs = 0.47, *p* = 0.01 | - | - | - | - | - | - | - | - | - | - | - |
| **OAT7** | - | - | Rs = 0.81, *p* = 0.004 | Rs = 0.76, *p* < 0.001 | Rs = 0.66, *p* < 0.001 | - | - | Rs = 0.44, *p* = 0.03 | - | Rs = 0.42, *p* = 0.01 | - | - | - | Rs = 0.43, *p* = 0.05 | - | - | - | Rs = 0.57, *p* = 0.02 | - |
| **OATP1A2** | - | - | - | Rs = 0.43, *p* = 0.04 | Rs = 0.52, *p* = 0.01 | - | Rs = 0.45, *p* = 0.04 | Rs = 0.84, *p* < 0.001 | - | - | Rs = 0.81, *p* = 0.02 | - | - | - | - | - | - | - | - |
| **OATP1B1** | Rs = 0.76, *p* = 0.009 | - | Rs = 0.64, *p* = 0.02 | Rs = 0.76, *p* < 0.001 | Rs = 0.62, *p* < 0.001 | - | Rs = 0.37, *p* = 0.03 | Rs = 0.39, *p* = 0.04 | - | - | - | - | - | - | - | - | - | - | - |
| **OATP1B3** | - | - | - | Rs = 0.63, *p* = 0.001 | Rs = 0.59, *p* = 0.002 | - | - | - | - | Rs = 0.73, *p* < 0.001 | - | - | - | - | - | - | - | - | - |
| **OATP2B1** | Rs = 0.7, *p* = 0.01 | - | Rs = 0.85, *p* < 0.001 | Rs = 0.73, *p* < 0.001 | Rs = 0.54, *p* < 0.001 | Rs = 0.35, *p* = 0.04 | - | Rs = 0.39, *p* = 0.04 | Rs = 0.38, *p* = 0.02 | - | - | - | - | - | - | - | - | Rs = 0.65, *p* = 0.002 | Rs = -0.83, *p* < 0.001 |

- denotes no correlation (*p* > 0.05).

**Supplementary Figures**


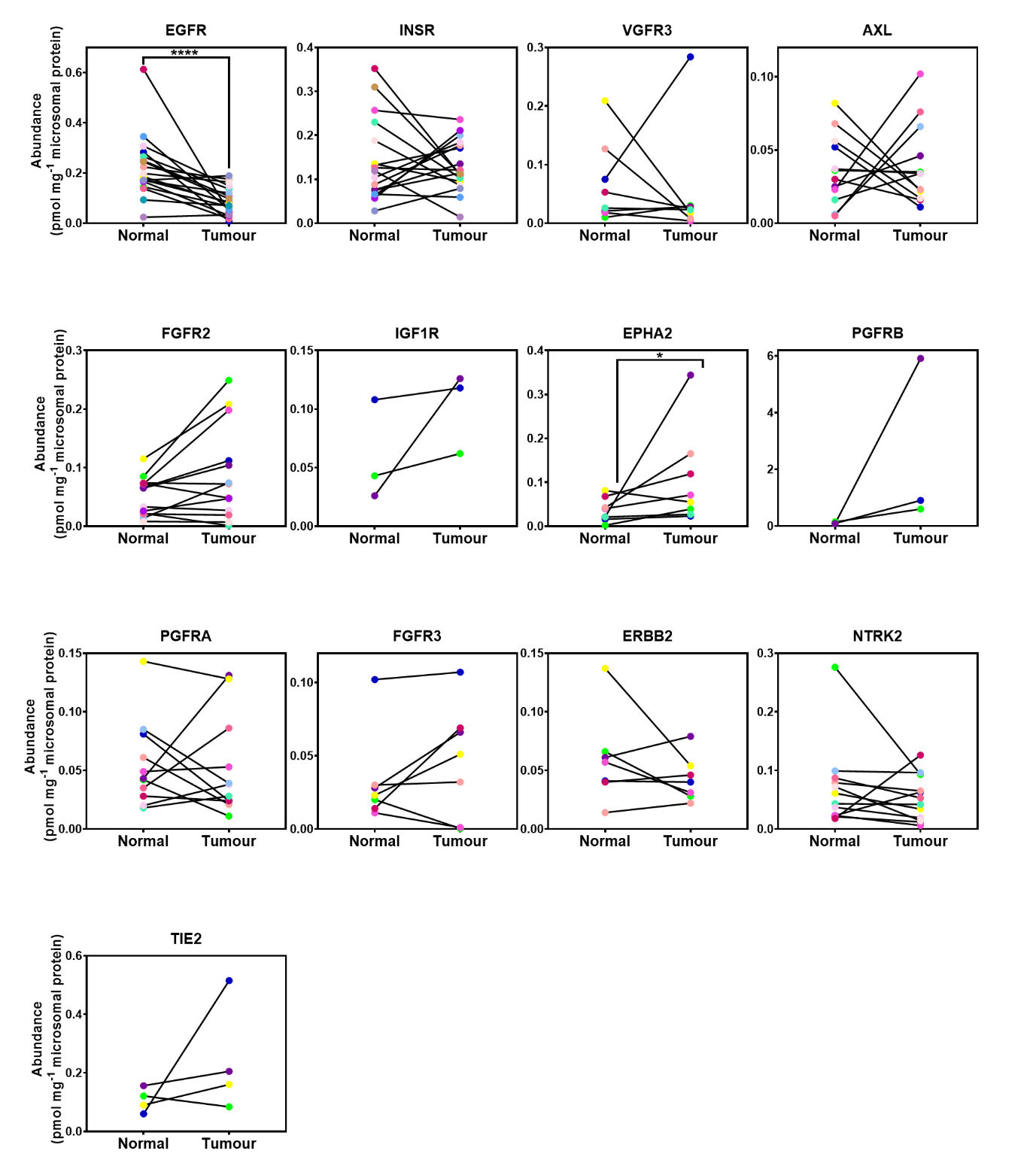


**Figure S1.** Absolute abundance of RTKs in paired (same donor) non-tumorous (histologically normal) and tumorous samples. Each line connects abundance in matched tissue samples. Wilcoxon test was used to assess statistically significant differences between non-tumorous (histologically normal) and matched tumorous samples for each protein; *p < 0.05, **** p < 0.0001.


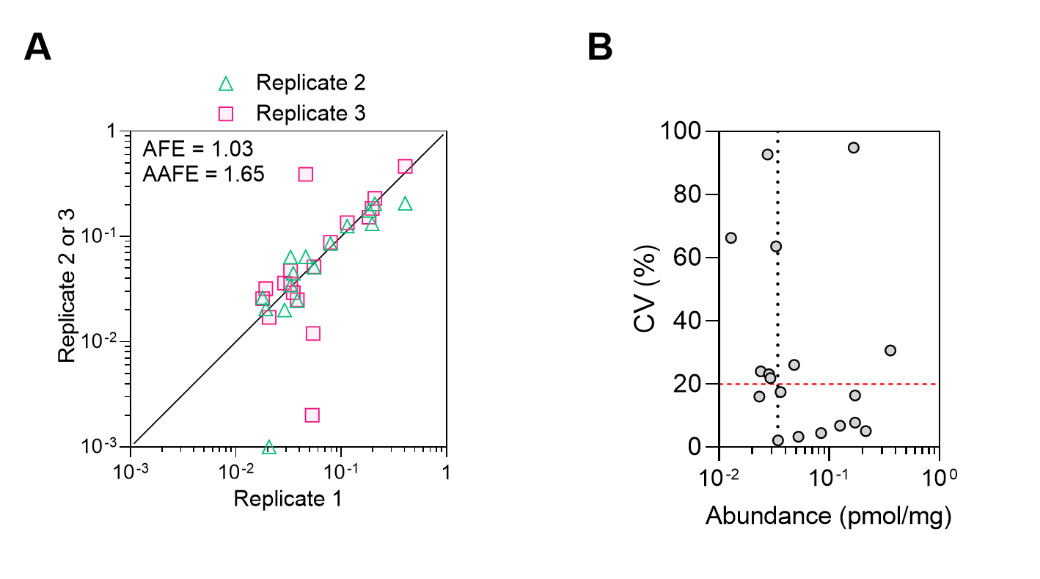


**Figure S2.** Technical evaluation of proteomic measurements. Precision (AFE) and accuracy (AAFE) of replicate measurements (A) and technical variability (B) of RTKs. Variability is represented by percent coefficient of variations (CV%) used to define the lower limit of quantification (LLOQ). AFE, average fold error; AAFE, absolute average fold error. The lower limit of quantification (LLOQ) was 0.03 pmol/mg microsomal protein.


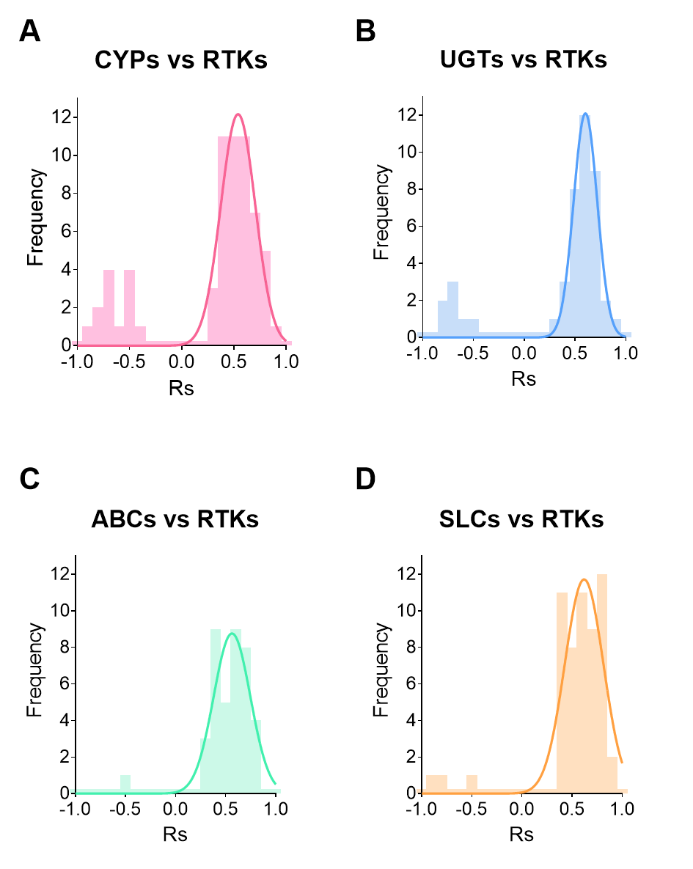


**Figure S3.** Distribution of frequency of statistically significant correlations (p < 0.05) between RTKs and CYPs (A), UGTs (B), ABC transporters (C) and SLCs (D). Rs is Spearman correlation coefficient.
